# Immunomodulatory Biomaterials for Bone and Soft Tissue Chronic Inflammation Diseases

**DOI:** 10.1002/smsc.202500260

**Published:** 2025-10-30

**Authors:** Yiming Li, Xudong Xie, Chong Ding, Shengming Zhang, Liangcong Hu, Bobin Mi, Mengfei Liu, Guohui Liu

**Affiliations:** ^1^ Department of Orthopedics Union Hospital Tongji Medical College Huazhong University of Science and Technology Wuhan 430022 China; ^2^ Hubei Province Key Laboratory of Oral and Maxillofacial Development and Regeneration Wuhan 430022 China; ^3^ Department of Plastic Surgery Zhongnan Hospital Wuhan University Wuhan 430071 China

**Keywords:** application of advanced materials, biomaterials, bone and soft tissue, chronic inflammation, immunomodulatory

## Abstract

Chronic inflammatory diseases of bone and soft tissue pose significant clinical challenges due to their complex pathogenesis and the limitations of conventional therapies, which often fail to address immune microenvironment dysregulation. This review explores the pivotal roles of key immune cells (including mast cells, macrophages, neutrophils, T cells, B cells, and dendritic cells) in driving inflammatory progression and tissue damage through dynamic cellular interactions and cytokine networks. It systematically analyzes the molecular and structural foundations of immunomodulatory biomaterials, such as nanoparticles, hydrogels, and scaffolds, which offer precise spatiotemporal control over immune cell phenotypes and inflammatory mediators. By integrating advances in immunology and materials science, this review highlights how surface functionalization, controlled drug release, and composite material strategies synergistically restore immune homeostasis and promote tissue regeneration. Studies across common chronic inflammatory diseases (e.g., osteoporosis, osteomyelitis, osteoarthritis, diabetic wounds, spinal cord injury, and intervertebral disc degeneration) demonstrate the therapeutic potential of biomaterial‐mediated immunomodulation, such as nanoparticle‐driven macrophage polarization, cytokine‐loaded hydrogel‐mediated immune cell balance, and scaffold‐guided immune cell recruitment. Challenges in clinical translation, including material biocompatibility and multicomponent synergy, are critically addressed. This review underscores the transformative potential of immunomodulatory biomaterials as next‐generation precision therapies to overcome therapeutic bottlenecks in chronic inflammatory diseases.

## Graphical abstract

This review analyzes immunomodulatory biomaterial strategies for treating chronic inflammatory disorders in bone and soft tissues. It explores emerging mechanisms for resolving inflammation while promoting tissue regeneration, offering insights for developing targeted immunotherapeutic approaches that bridge material science with immunology for improved disease management.

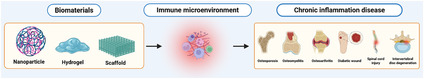

## 1 Introduction

Chronic inflammatory diseases of bone and soft tissue have become a major challenge in global health due to their complex immunopathological mechanisms and the limitations of traditional therapeutic approaches. The core pathological feature of these diseases is the dynamic imbalance of the immune microenvironment, in which key immune cells including mast cells, macrophages, neutrophils, T cells, B cells, and dendritic cells drive tissue damage through complex interaction networks.^[^ ^1^, ^2^ ^]^ For example, M1/M2 macrophage polarization imbalance, Th17/Treg ratio dysregulation, and pro‐inflammatory/anti‐inflammatory factor network disruption all play indispensable roles in disease progression.^[^ ^3^, ^4^, ^5^ ^]^

Compared with the breakthrough advances achieved in the field of tumor immunotherapy through PD‐1/PD‐L1 (programmed death 1/programmed death‐ligand 1) inhibitors and chimeric antigen receptor (CAR) T cell therapy,^[^ ^6^, ^7^ ^]^ immunomodulatory strategies for chronic inflammatory diseases remain relatively lagging. This disparity reflects the important difference in therapeutic objectives between the two: Tumor therapy requires enhancing immune responses, while chronic inflammation repair needs to achieve a balance between suppressing excessive inflammation and activating tissue regeneration. Although new drugs such as Interleukin (IL)‐6 receptor antagonists (e.g., tocilizumab) and the Janus kinase (JAK) inhibitors have been applied clinically,^[^ ^8^, ^9^ ^]^ their mechanisms of action remain limited to neutralizing inflammatory factors and have not yet achieved systematic remodeling of the immune microenvironment.

Immunomodulatory biomaterials, as emerging therapeutic platforms, demonstrate unique advantages.^[^ ^10^, ^11^ ^]^ The core advantages of these materials include: 1) achieving multidimensional regulation of the immune microenvironment through the physicochemical properties of the materials themselves or loaded bioactive molecules; 2) utilizing surface functionalization strategies to achieve precise targeting of specific immune cells; 3) maintaining effective therapeutic concentrations at lesion sites through controlled release systems; and 4)integrating physical properties and biochemical signals of materials to synergistically regulate immune responses.^[^ ^12^ ^]^

Specifically, nanoparticles can influence immune function by regulating macrophage polarization and reprogramming.^[^ ^13^ ^]^ These nanocarriers can be selectively taken up by specific immune cells based on differences in size, surface charge, and modification groups, subsequently altering cell phenotypes through delivery of immunomodulatory molecules or direct material‐cell interactions. Hydrogel systems achieve sustained release of bioactive molecules within 3D network structures by loading cytokines or immune signaling pathway agonists, dynamically regulating local immune cell balance.^[^ ^14^ ^]^ Their tunable mechanical properties and degradation characteristics further influence immune cell migration, proliferation, and differentiation. Three‐dimensional scaffolds not only provide structural support but also influence immune cell recruitment patterns and functional differentiation by optimizing parameters such as pore size, porosity, surface topography, and chemical functional groups.^[^ ^15^ ^]^

However, current research still faces numerous challenges. Most material designs focus on single immune targets, failing to fully consider the multicellular and multifactorial complexity of the chronic inflammatory microenvironment. The temporal matching between material degradation kinetics and immunomodulatory effects requires optimization. Additionally, factors such as differences between animal models and human immune systems, potential immunogenicity of materials, and the effects of complex in vivo environments on material performance all constrain the clinical translation of immunomodulatory biomaterials.

This review will systematically explore: 1) the functional characteristics and interaction mechanisms of key immune cells in chronic inflammation; 2) the molecular and structural basis for immune response regulation by biomaterials such as nanoparticles, hydrogels, and scaffolds; 3) immunomodulatory therapeutic strategies for common chronic inflammatory diseases of bone and soft tissue. Through in‐depth analysis of the synergistic effects of composite material systems in reshaping the immune microenvironment and promoting tissue regeneration, this review aims to provide theoretical foundations for developing next‐generation precision immune engineering therapeutic approaches and drive breakthrough advances in the treatment of chronic inflammatory diseases.

## 2 Immune Cells

### 2.1 Mast Cell

Mast cells (MCs) are innate immune cells belonging to the bone marrow lineage, and are found in almost all vascularized tissues. It is generally believed that mast cells in both human and rodents are derived from hematopoietic stem cells (HSCs), which are then grown into myeloid progenitor cells in bone marrow and then differentiated into mast cell progenitor cells (MCPs).^[^ ^16^ ^]^ Ultimately, immature MCPs were released into the blood and recruited to various target organs based on surface molecules such as α4β7 integrin, mucosal addressing cell adhesion molecule‐1 (MAdCAM‐1), and vascular cell adhesion molecule‐1 (VCAM1) to closely contact with the microenvironment and some vascularized organs.^[^ ^17^, ^18^ ^]^ Meanwhile, specific factors in these tissues, including cytokines, growth factors, and extracellular matrix, stimulated phenotypic maturation of MCPs, which then performed different functions.^[^ ^19^ ^]^ In humans, mast cells are classified into MC_TC_s (predominantly distributed in the skin, gastrointestinal tract, and conjunctiva) and MC_T_s (predominantly distributed in the lungs, nose, and sinuses) based on whether they secrete chymase.^[^ ^20^, ^21^ ^]^ It has been reported that mast cells regulate both acute and chronic inflammation. Several cytokines, chemokines and growth factors produced by mast cells, including chemokine ligand‐1 (CCL1), CCL4, tumor necrosis factor (TNF), histamine, interleukin 4 (IL‐4), IL‐13, IL‐8, monocyte chemoattractant protein‐1 (MCP‐1), and macrophage inflammatory protein‐1 (MIP‐1), widely recruited more white blood cells and monocytes from the blood, and further induced differentiation of monocytes into macrophages and dendritic cells.^[^ ^22^, ^23^, ^24^ ^]^ Additionally, mast cells also involved in inflammation process via interaction with macrophages, T cells and Treg cells.

### 2.2 Macrophage

Circulating blood monocytes are recognized as macrophage precursors.^[^ ^25^ ^]^ These cells originate from hematopoietic stem cells (HSCs) in bone marrow, with committed monocyte progenitors (cMops) entering the bloodstream following sequential developmental phases: common myeloid progenitor (CMP), granulocyte‐macrophage progenitor (GMP), and macrophage‐dendritic cell precursor (MDP) stages.^[^ ^26^ ^]^ Nevertheless, macrophage populations exhibit dual origins, as embryonic yolk sac‐derived precursors generate most tissue‐resident macrophages independently of monocytic differentiation.^[^ ^27^, ^28^ ^]^ Numerous tissues maintain coexisting populations of embryonically derived and monocyte‐originated macrophages that execute distinct functional roles. These immune cells safeguard organisms through four fundamental mechanisms: environmental sensing, directional migration, pathogenic engulfment, and injury resolution, alongside adaptive immune modulation, cellular debris clearance, and postdamage tissue regeneration. Research demonstrates that macrophages display exceptional adaptability, with microenvironmental cues driving phenotypic shifts across a spectrum from pro‐inflammatory (M1) to anti‐inflammatory/pro‐resolution (M2) states.^[^ ^29^ ^]^ Following removal of activating signals, macrophages typically transition toward pro‐resolution phenotypes favoring tissue restoration and inflammation resolution. The classical M1 activation pathway is primarily triggered by interferon‐γ (IFN‐γ), TNF‐α, or microbial components like lipopolysaccharide (LPS), while alternative M2 polarization occurs through diverse stimuli including IL‐4, IL‐13, and IL‐10,^[^ ^30^ ^]^ further categorized into four subsets: M2a (activated by IL‐4/IL‐13), M2b (induced by immune complexes/TLR ligands/IL‐1R agonists), M2c (stimulated by glucocorticoids/IL‐10), and M2d (activated via TLR ligands).^[^ ^31^, ^32^ ^]^ Although M1 and M2 subtypes broadly represent inflammatory versus anti‐inflammatory functions, both play context‐dependent roles in disease pathogenesis. Typically, M1 macrophages drive Th1‐type immune responses by secreting pro‐inflammatory mediators, combating intracellular pathogens, and mediating tissue destruction.^[^ ^33^, ^34^, ^35^ ^]^ Phenotypic switching from M1 to M2 states is regulated by IL‐13/IL‐4 from mast cells, basophils, and Th2 lymphocytes. M2 macrophages support Th2 responses, cellular debris removal, tissue reconstruction, and wound closure,^[^ ^36^, ^37^ ^]^ while paradoxically facilitating tumor advancement, blood vessel formation, and bone resorption processes.^[^ ^38^, ^39^, ^40^ ^]^ Functional diversity persists among M2 subtypes: M2a cells abundantly produce IL‐1 receptor antagonists (IL1RN), while M2c cells display potent anti‐inflammatory effects via IL‐10 and TGF‐β secretion. The M2b subset uniquely co‐releases anti‐inflammatory (IL‐10) and pro‐inflammatory (IL‐1β, IL‐6, TNF‐α) cytokines. M2d macrophages activated through TLR agonists and ADORA2A signaling suppress pro‐inflammatory cytokine output while enhancing IL‐10 and vascular endothelial growth factor (VEGF) expression.^[^ ^41^ ^]^ Thus, the M1/M2 balance contributed to maintaining homeostasis of the immune microenvironment, rather than transforming macrophages into a single anti‐inflammatory functional phenotype.^[^ ^42^ ^]^

### 2.3 Neutrophil

Neutrophils are myeloid white blood cells that make up 50%–70% of the total number of human peripheral blood white blood cells and are one of the major players during acute inflammation.^[^ ^43^ ^]^ As first responders, neutrophils are the initial host defense system against a variety of pathogens, including bacteria, fungi, and protozoa.^[^ ^44^ ^]^ Neutrophils are continuously produced in the bone marrow (up to 2 × 10^11^ cells per day), and they mature from myeloblasts and eventually develop intopolymorphonuclear segmented cells.^[^ ^45^ ^]^ The primary regulator of this developmental process is granulocyte colony‐stimulating factor (G‐CSF), and lack of G‐CSF receptor causes extreme neutropenia in mice and humans.^[^ ^46^ ^]^ The cytokines IL‐6, IL‐4, and granulocyte‐macrophage colony‐stimulating factor (GM‐CSF) also promote granulocyte production in vivo.^[^ ^44^ ^]^ The recruitment cascade of neutrophils includes tethering, rolling, adhesion, crawling and transendothelial migration.^[^ ^47^ ^]^ Inflammatory mediators (such as leukotrienes, cytokines, and histamine) stimulate endothelial cell surface changes.^[^ ^47^ ^]^ When vascular endothelial cells are activated, the expression of P‐selectin and E‐selectin is significantly upregulated. These molecules act as “molecular hooks” to capture rapidly flowing neutrophils from the bloodstream, initiating their rolling along the vessel wall.^[^ ^48^, ^49^ ^]^ This rolling process slows down neutrophil movement, creating conditions for subsequent stable adhesion. Notably, L‐selectin mediates a unique cascade reaction: circulating neutrophils can bind to neutrophils already rolling on the vessel wall through L‐selectin, forming a “secondary capture” phenomenon that amplifies neutrophil recruitment.^[^ ^50^ ^]^ Upon stimulation by chemokines such as IL‐8, neutrophils become activated, and their surface integrin molecules undergo a conformational change from a low‐affinity to a high‐affinity state. This conformational change enables neutrophils to crawl stably on the endothelial surface and ultimately transmigrate across the vessel wall to reach the site of inflammation.^[^ ^51^ ^]^ As part of the first line of defense against microorganisms, neutrophils contain a range of antimicrobial effecting functions, including phagocytosis, degranulation, and formation of neutrophilic extracellular traps (NETs). Neutrophils are also involved in regulating adaptive immunity, such as the function of B cells and T cells. Neutrophils are considerable producers of B cell activating factor and proliferation‐inducing ligand, both of which are necessary for B cell survival and its activation.^[^ ^52^ ^]^ Studies have shown that during IFN‐γ stimulation, neutrophils upregulate levels of their major histocompatibility complex class II (MHC‐II) and costimulatory molecules, promoting Th1 and Th17 differentiation.^[^ ^53^ ^]^

### 2.4 Lymphocyte

#### 2.4.1 T Cell

Conventional T cells express highly diverse T cell receptors (TCR), composed of α and β chains, capable of recognizing corresponding antigens. Based on their co‐receptor expression, T cells are further divided into CD4+ and CD8+ T cells, where naive CD4+ αβ T cells can differentiate into various specialized helper T cell subsets (Th1, Th2, Th17, Th9, Th22, follicular Th cells, or regulatory T cells (Treg)) under specific cytokine environments.^[^ ^54^ ^]^ Generally, T cells and their subsets play important roles in tissue repair and regeneration following macrophages. However, the accumulation levels of T cells at injury sites vary greatly among different tissues and are not fully understood. In recent years, the role of αβ T cells in tissue regeneration has attracted widespread attention. Studies have revealed that these T cells can be broadly divided into two functionally opposing subsets: those that promote tissue regeneration and those that inhibit tissue regeneration. Notably, tissue‐resident αβ T cells, which are T cell populations that permanently reside in specific tissues, predominantly exhibit proregenerative functional characteristics.^[^ ^55^, ^56^ ^]^ Conventional T cells (CD8+ and CD4+ T cells) typically possess pro‐inflammatory activity due to their expression and secretion of tumor necrosis factor‐α (TNF‐α) and TNF‐γ,^[^ ^57^, ^58^ ^]^ while the pro‐inflammatory effects of conventional T cells can be suppressed through various mechanisms by FOXP3‐expressing Treg cells within the classical CD4+ T cell subset, which simultaneously produce anti‐inflammatory cytokines such as IL‐10, transforming growth factor‐β (TGF‐β), and IL‐35.^[^ ^56^, ^58^, ^59^ ^]^ Furthermore, Tregs can also regulate the activities of neutrophils, inflammatory macrophages, as well as CD4+ and CD8+ T cells, promoting tissue repair and regeneration, though these mechanisms may be tissue‐specific.^[^ ^56^, ^57^, ^58^ ^]^

#### 2.4.2 B Cell

B cells mature in the bone marrow, the central immune organ. The major events in the differentiation and development of B cells in central immune organs are the expression of functional B cell receptor (BCR) and the formation of B cell autoimmune tolerance. Cytokines and adhesion molecules expressed in the bone marrow microenvironment, especially stromal cells, play a key role in inducing the differentiation and development of B cells. Naive B cells differentiate into memory B cells (MBCs) and plasma cells (PCs) during germinal center (GC) reactions.^[^ ^60^ ^]^ In addition to IgG and IgA memory B cells, about 50% of peripheral blood memory B cells express IgM, with or without IgD.^[^ ^61^ ^]^ Plasma cells are the main producers of antibodies during and after infection.^[^ ^62^ ^]^ As essential components of adaptive immunity, B cells are capable of producing a diverse repertoire of antibodies and secreting proinflammatory cytokines to facilitate the clearance of foreign antigens and cancer cells. Additionally, B cells have been shown to play a role in immune regulation by suppressing excessive inflammatory responses via cell–cell contact mechanisms and/or through the secretion of anti‐inflammatory cytokines, such as IL‐10, IL‐35, and TGF‐β.^[^ ^63^ ^]^

### 2.5 Dendritic Cell

Dendritic cells (DCs) are the most effective antigen‐presenting cells with many dendritic processes at maturity, which can recognize, ingest and process exogenous antigens, and present antigenic peptides to initial T cells to induce T cell activation and proliferation.^[^ ^64^ ^]^ DC is the initiator of the body's adaptive immune response and also the “bridge” connecting the innate immune response and the adaptive immune response. The main types of DC include conventional DC (cDC), which can be divided into various subtypes, plasmacytoid DC (pDC), and inflammatory DC (infDC), which are involved in the induction and initiation of different immune responses.^[^ ^65^ ^]^ DC expresses a variety of pattern recognition receptors (PRR, such as mannose receptors, toll‐like receptors (TLRs)) and Fc receptors to recognize antigens and takes up antigens through pinocytosis, phagocytosis, and receptor‐mediated endocytosis. DC affects the differentiation of CD4+ T cells and plays a major role in regulating initial CD4+ T cell proliferation. DC activates initial CD4+ T lymphocytes through a series of coordinated signals, including antigen presentation of major histocompatibility complex (MHC) molecules, costimulatory signals directing cell surfaces, and cytokine signals.^[^ ^66^ ^]^ DC can secrete a variety of cytokines and chemokines to regulate the function of other immune cells. For example, DC secretes a large amount of IL‐12 to induce the differentiation of initial T cells (Th0) into Th1 cells.

**Figure 1:**
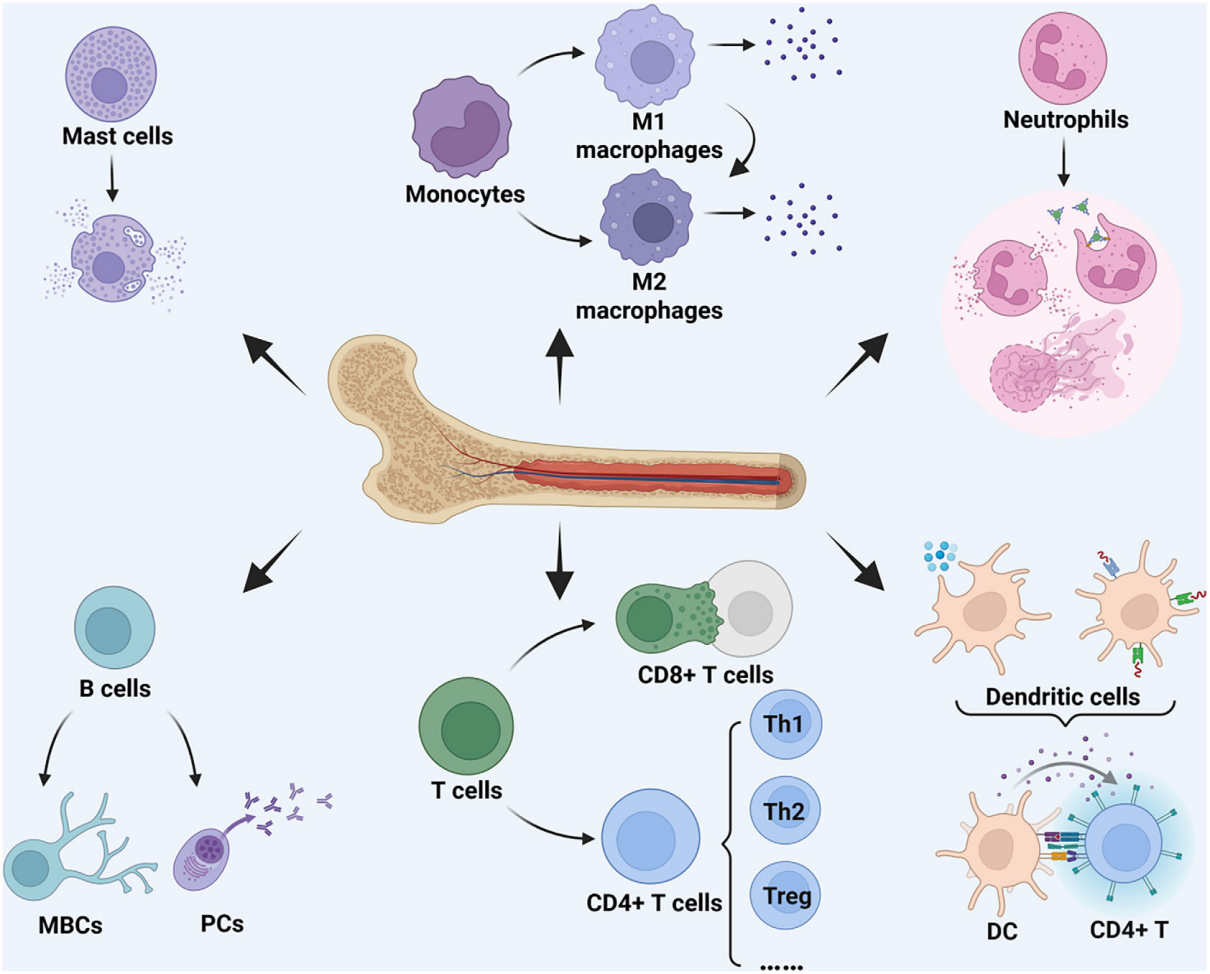
Main immune cells in chronic inflammatory diseases. Mast cells originating from hematopoietic stem cells activate and produce a variety of cytokines, chemokines and growth factors. Dynamic regulation of M1/M2 polarization of macrophages derived from monocyte differentiation. Neutrophils derived from bone marrow activate the inflammatory response through phagocytosis, degranulation and formation of NETs. Different subsets of T cells produce different pro‐inflammatory and anti‐inflammatory effects (such as CD8+T, Th1, Th2, Treg, etc.). B cells can differentiate into memory B cells (MBCs) and plasma cells (PCs), produce a variety of antibodies, and secrete pro‐inflammatory cytokines. Dendritic cells (DCs) participate in antigen presentation and regulate immune cell function in a variety of ways, such as phagocytosis or receptor‐mediated endocytosis. Created in https://BioRender.com.

The complex interplay among these immune cells creates a dynamic inflammatory microenvironment that presents both challenges and opportunities for therapeutic intervention (**Figure** **1**). Understanding the specific functions and interactions of mast cells, macrophages, neutrophils, T cells, B cells, and dendritic cells lays the foundation for designing immunomodulatory biomaterials with targeted therapeutic effects. The key to successful biomaterial‐based immunotherapy lies in harnessing these cellular mechanisms—whether by promoting macrophage polarization from M1 to M2 phenotype, modulating T cell differentiation, suppressing excessive neutrophil activation, or enhancing regulatory immune responses.

Modern biomaterial design strategies fully leverage this immune cell knowledge by integrating specific molecular cues, physical properties, and controlled release mechanisms to precisely influence cellular behavior. For instance, biomaterials can be engineered to present surface ligands that selectively bind to immune cell receptors, deliver cytokines that shift inflammatory balance, or create microenvironments that favor tissue repair over chronic inflammation. This transition from understanding immune cell biology to applying biomaterial engineering represents a paradigm shift in treating chronic inflammatory diseases, moving from broad immunosuppression to sophisticated immunomodulation that works in harmony with the body's natural healing processes.

## 3 Immune System Modulated by Biomaterials

**Figure 2:**
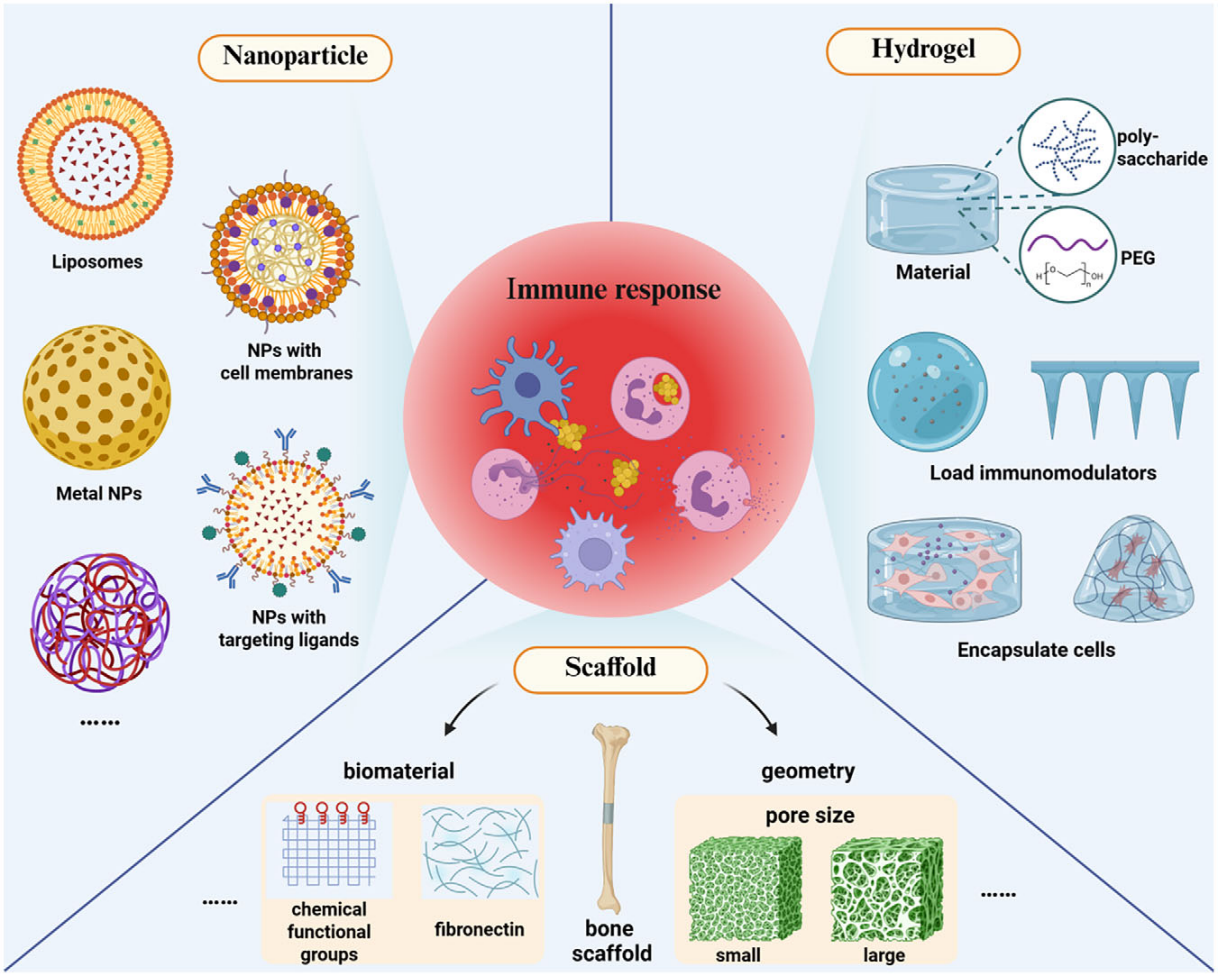
Major immunomodulatory biomaterials. The main strategies of nanoparticle targeting to regulate inflammatory response include the preparation of targeted ligand of simulated cell surface proteins, cell membrane encapsulation, liposomes, metal materials, etc. The hydrogels can regulate the immune system by means of immunomodulatory components in the materials, loading immunomodulators and encapsulating immune cells. Immune regulation of scaffolds can be modulated by applying biological materials such as fibronectin and chemical functional groups modification, and changing geometric properties such as pore size. Created in https://BioRender.com.

In the field of tissue repair and regeneration, biomaterial systems represented by nanoparticles, hydrogels and 3D scaffolds have demonstrated unique advantages (**Figure** **2**). Due to the heterogeneity of these materials respectively, they will all have specific interactions with the host immune system. This crucial biological regulatory response will directly affect the therapeutic efficacy and tissue remodeling process of the materials.

### 3.1 Nanoparticle

Since nanoparticles (NPs) gained clinical approval as novel diagnostic and therapeutic platforms, they have demonstrated revolutionary potential in precision medicine.^[^ ^67^ ^]^ This breakthrough stems from the precise control capabilities of nanotechnology at the microscale—through systematic optimization of key parameters such as particle size distribution, geometric configuration, and surface properties, controlled design of nanoparticle performance has been achieved.^[^ ^68^ ^]^ At the molecular engineering level, researchers have successfully constructed intelligent nanosystems with both tissue‐specificity and functional‐specificity through precise control of nanomaterial assembly, surface functional group modification, and targeted ligand conjugation.^[^ ^69^, ^70^ ^]^

The biological effects of nanoparticles are closely related to their in vivo behavior. After entering target tissues, the interactions between nanoparticles and endogenous biomolecules directly influence their therapeutic efficacy and safety.^[^ ^71^ ^]^ These interactions endow nanoparticles with unique dual immunomodulatory functions: They can activate immune responses through the physicochemical properties of the materials themselves, while also achieving precise inflammatory regulation through drug‐loading capabilities. Notably, certain nanoparticles can selectively activate specific immune effector components, providing novel strategies for vaccine adjuvant development and targeted immunotherapy.^[^ ^72^, ^73^ ^]^

In response to the complexity of the inflammatory immune microenvironment, researchers have developed various functionalized nanoparticle systems.^[^ ^74^ ^]^ Currently, these mainly include: 1) targeted nanoparticles with surface‐modified biomimetic ligands; 2) biomimetic nanoparticles coated with cell membranes; 3) liposomal nanoparticles with excellent biocompatibility;^[^ ^75^ ^]^ and 4) metal and metal oxide nanoparticles with unique physicochemical properties.^[^ ^76^ ^]^ These diverse nanoplatforms provide rich technological options for the precision diagnosis and treatment of inflammatory diseases (**Table** **1**).

**Table 1:** Various functionalized nanoparticle systems and their immunomodulatory mechanisms.

Nanoparticle type	Specific materials	Immunomodulatory mechanism	Reference
Surface‐modified biomimetic ligand‐targeted nanoparticles	IgG‐modified bilirubin/JPH203 self‐assembled nanoparticles (IgG/BRJ NPs)	Promote M1 macrophage recognition and phagocytosis, inhibit inflammation	[83]
	Ulva lactuca polysaccharide‐modified selenium nanoparticles (ULP‐SeNPs)	Inhibit NF‐κB nuclear translocation, regulate IFN‐α and IL‐6, suppress macrophage activation	[82]
Cell membrane‐coated biomimetic nanoparticles	Biomimetic DNase I delivery system	Mimic inflammatory chemotaxis, clear NETs, protect lung tissue	[81]
Biocompatible liposome nanoparticles	F4/80‐conjugated lipid nanoparticles	Targeted delivery of TAK1‐specific siRNA to suppress the pro‐inflammatory macrophage phenotype	[84]
Metal and metal oxide nanoparticles	Silver nanoparticles (AgNPs)	Induce macrophage secretion of TNF‐α and IL‐6, promote M1 polarization	[77]
	SOD@HMUiO‐MnTCPP nanoparticles (S@H@M NPs)	Integrate natural and artificial enzymes, efficiently scavenge ROS	[85]
Polymer nanoparticles	Self‐assembled glyco‐nanoparticles (glyco‐NPs)	Induce macrophage M2‐to‐M1 polarization, upregulate CD86, and increase cytokine secretion	[78]
	Carboxyl‐modified polyurethane nanoparticles (PU NPs)	Inhibit M1 polarization, reduce TNF‐α and IL‐1β secretion	[79]
	Photothermal‐responsive polymer micelles (PPIR780‐ZMS)	Enhance ROS generation, activate CD8+ and CD4+ T cell expansion and infiltration	[80]

Nanoparticles exert immunomodulatory effects by regulating immune cell functions. In terms of macrophage polarization regulation, different nanomaterials exhibit unique immunomodulatory properties. Silver nanoparticles (AgNPs) induce RAW264.7 macrophages to secrete pro‐inflammatory cytokines such as TNF‐α and IL‐6 more strongly than gold nanoparticles (AuNPs), promoting M1‐type polarization.^[^ ^77^ ^]^ Conversely, self‐assembled glyco‐nanoparticles (glyco‐NPs) developed by Su et al. can effectively induce macrophage transformation from immunosuppressive M2 to pro‐inflammatory M1 phenotype, characterized by upregulated CD86 expression and increased pro‐inflammatory cytokine secretion.^[^ ^78^ ^]^ In contrast, carboxyl‐modified polyurethane nanoparticles (PU NPs) inhibit M1 polarization and reduce the secretion of TNF‐α and IL‐1β.^[^ ^79^ ^]^ In terms of adaptive immune regulation, Li et al. designed polymer micelles (PP_IR780_‐ZMS) assembled from IR780 dye and manganese zinc sulfide nanoparticles (ZMS), which effectively promoted the expansion and infiltration of CD8+ and CD4+ T cells through enhanced reactive oxygen species (ROS) generation, thereby enhancing antitumor immune responses.^[^ ^80^ ^]^

The drug delivery function of nanoparticles provides novel strategies for precise inflammatory treatment. Targeting neutrophil extracellular traps (NETs) in acute lung injury, researchers developed a biomimetic DNase I delivery system that enhances pulmonary targeting by mimicking inflammatory chemotaxis, effectively clearing NETs and protecting lung tissue.^[^ ^81^ ^]^ In inflammatory bowel disease treatment, Ulva lactuca polysaccharide‐modified selenium nanoparticles (ULP‐SeNPs) significantly alleviate acute colitis by inhibiting nuclear factor‐kappaB (NF‐κB) nuclear translocation and macrophage activation.^[^ ^82^ ^]^ For osteoarthritis, an immunoglobulin G‐conjugated bilirubin/JPH203 self‐assembled nanoparticle (IgG/BRJ NP) leverage the high phagocytic capacity of M1 macrophages to achieve targeted anti‐inflammation.^[^ ^83^ ^]^ In studies on viral pneumonia, conjugating the macrophage‐specific antibody F4/80 to lipid nanoparticles (LNPs) enabled targeted delivery of siRNA against TAK1, an upstream kinase in inflammatory signaling. This approach significantly suppressed the pro‐inflammatory macrophage phenotype and reduced inflammation in vitro and in vivo, demonstrating the efficacy and translational potential of macrophage‐targeted interventions.^[^ ^84^ ^]^ Additionally, Li et al. spatially organized natural superoxide dismutase (SOD) and artificial enzymes within zirconium‐based macroporous mesoporous metal‐organic frameworks (MOFs) to construct cascade antioxidant nanoparticles (S@M@H NPs) with highly efficient ROS scavenging capability, and demonstrated their ability to significantly promote diabetic wound healing.^[^ ^85^ ^]^

These studies demonstrate that through precise design of the physicochemical properties and functional modifications of nanoparticles, precise regulation of immune responses can be achieved, providing diverse nanomedicine strategies for the treatment of inflammatory diseases.

### 3.2 Hydrogel

**Figure 3:**
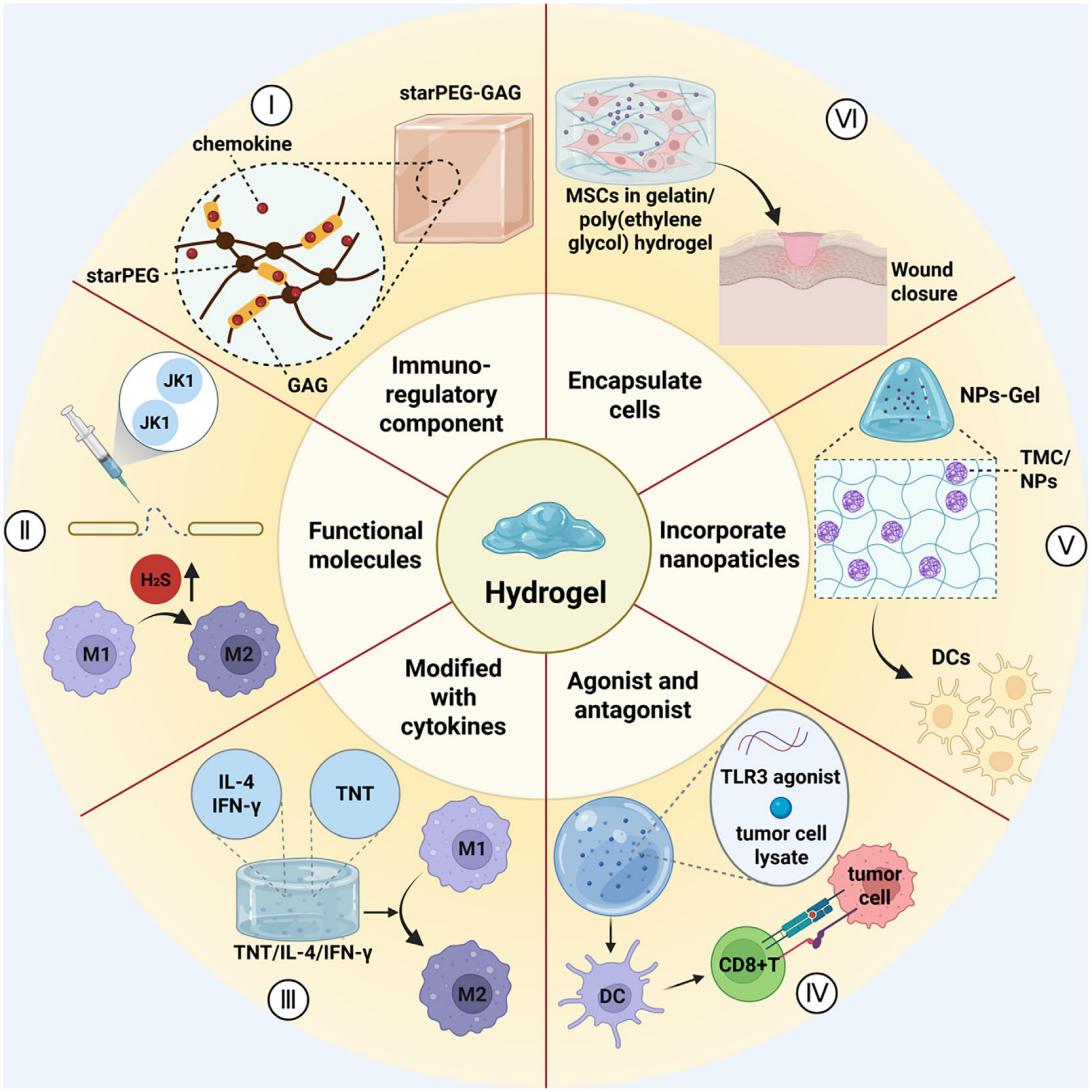
Immunomodulatory effects of hydrogels. I) Immunoregulatory components: star PEG‐GAGs removes pro‐inflammatory factors through strong electrostatic interaction.^[^ ^95^ ^]^ II) Functional molecules: HA‐JK1 hydrogel promotes M2 transformation and reduces inflammation by enhancing the release of hydrogen sulfide.^[^ ^99^ ^]^ III) Cytokines: Complex hydrogels composed of TNT/IL‐4/IFN‐γ promote the transformation of macrophage M1 phenotype to M2 phenotype.^[^ ^102^ ^]^ IV) Agonist and antagonist: Encapsulated TLR3 agonists and tumor antigen suppress tumors by activating DCs to trigger cytotoxic T lymphocyte immune responses.^[^ ^103^ ^]^ V) Nanoparticles: Inclusion of TMC/NPs into HA hydrogels promotes DCs maturation.^[^ ^106^ ^]^ VI) Cells: Gelatin/PEG hydrogel biomatrix containing MSCs promotes wound healing.^[^ ^107^ ^]^ Created in https://BioRender.com.

Hydrogels demonstrate significant advantages in the biomedical field due to their unique physicochemical properties. Their excellent biocompatibility, hydrophilicity, and three‐dimensional porous structure confer intelligent drug controlled‐release capabilities and soft tissue‐like elasticity.^[^ ^86^, ^87^ ^]^ Based on their morphological plasticity, hydrogels can be processed into various forms including films, fiber scaffolds, injectable formulations, and microneedle patches, finding widespread applications in novel dressing and tissue adhesive development.^[^ ^88^, ^89^ ^]^ In wound management, while the high water content of hydrogels limits their ability to absorb exudates, they can maintain a moist healing environment through active rehydration, avoiding wound dehydration caused by traditional dressings.^[^ ^90^, ^91^ ^]^ Their physical cooling effect can also reduce wound temperature and alleviate inflammatory responses. Meanwhile, the 3D network structure of hydrogels resembles natural extracellular matrix (ECM), providing an ideal microenvironment for cell growth.^[^ ^92^ ^]^ By modulating crosslinking density and pore size distribution, they can mimic the mechanical properties of different tissue.^[^ ^93^ ^]^ Notably, to assess and optimize the risks of chronic inflammation and fibrosis, studies have established an in vivo high‐throughput screening platform by implanting cell‐barcoded alginate systems in mice and nonhuman primates, enabling comparison of the antifibrotic properties of different hydrogel formulations and the intensity of host responses.^[^ ^94^ ^]^ With their dynamic responsiveness and bioactive molecule delivery capabilities, hydrogels have achieved significant progress in regulating immune cell behavior and promoting tissue regeneration, establishing themselves as a frontier field in immunomodulatory biomaterial research (**Figure** **3**).

Hydrogels can directly exert immunomodulatory effects through their structural design. For example, star‐shaped polyethylene glycol‐glycosaminoglycan (starPEG‐GAGs) hydrogels, leveraging their unique molecular structure, can efficiently capture various inflammatory chemokines (MCP‐1, IL‐8, MIP‐1α, and MIP‐1β), significantly reducing inflammatory responses and promoting tissue repair in chronic wound treatment.^[^ ^95^ ^]^ Natural polysaccharide hydrogels also demonstrate excellent immunomodulatory properties: Bletilla striata polysaccharide (BSP) hydrogels not only reduce levels of inflammatory factors such as TNF‐α but also upregulate epidermal growth factor (EGF) expression, accelerating wound healing;^[^ ^96^ ^]^ zwitterionic dextran hydrogels create a microenvironment favorable for tissue repair through multiple mechanisms including reactive oxygen species (ROS) scavenging, antiprotein adsorption, and inhibition of bacterial adhesion.^[^ ^97^ ^]^

Incorporating specific immunomodulatory molecules into hydrogels through physical adsorption or chemical conjugation enables more precise immune regulation. Carboxymethyl chitosan‐thioketone (CMCTS‐Tk) hydrogels loaded with curcumin not only protect cells from oxidative damage but also induce macrophage polarization toward the anti‐inflammatory M2 phenotype.^[^ ^98^ ^]^ Hyaluronic acid‐JK1 peptide hydrogel achieves intelligent regulation of the immune microenvironment through pH‐responsive release of hydrogen sulfide (H_2_S), enabling in situ polarization of M2 macrophages.^[^ ^99^ ^]^

The synergistic effects of multiple cytokines further expand hydrogel functionality. Gelatin hydrogels simultaneously loaded with two chemokines, IL‐8 and MIP‐3α, significantly promote angiogenesis and granulation tissue formation through cooperative immune cell recruitment.^[^ ^100^ ^]^ Polyvinyl alcohol/chitosan (PVA/CS) composite hydrogels loaded with stromal cell‐derived factor 1 (SDF‐1) can rapidly recruit bone marrow mesenchymal stem cells to participate in tissue repair.^[^ ^101^ ^]^ A more sophisticated design involves bilayer hydrogel systems that regulate the sequential release of IFN‐γ and IL‐4 through titanium nanotubes, achieving dynamic transformation of macrophage phenotypes.^[^ ^102^ ^]^

Targeting specific immune signaling pathways represents a more sophisticated design concept. Poly(L‐valine) hydrogels embedding tumor cell lysates and the TLR3 agonist poly(I:C) create an efficient in situ tumor vaccine system that potently activates dendritic cells and induces cytotoxic T lymphocyte responses.^[^ ^103^ ^]^ N,N,N‐trimethyl chitosan (TMC) hydrogels loaded with the PD‐1/PD‐L1 blocking peptide OPBP‐1 (oral PD‐L1 binding peptide 1) significantly enhance the anti‐tumor activity of CD8+ T cells by releasing immune checkpoint inhibition.^[^ ^104^ ^]^

The integration of nanoparticles with hydrogels endows materials with enhanced functionality. Gelatin‐based hydrogels loaded with folate‐functionalized polydopamine nanoparticles (FA‐PDA@NPs) achieve dual anti‐inflammatory and chondroprotective effects in rheumatoid arthritis treatment by targeted delivery of leonurine and downregulation of the JAK2/STAT3 signaling pathway.^[^ ^105^ ^]^ Hyaluronic acid hydrogels embedding TMC nanoparticles loaded with ovalbumin create a vaccine delivery system with adjuvant effects, where the sustained‐release properties significantly enhance immune responses.^[^ ^106^ ^]^

Encapsulating immunomodulatory living cells within hydrogels enables continuous in situ therapy. Mesenchymal stem cells (MSCs) embedded in gelatin/PEG hydrogels effectively suppress foreign body reactions and promote tissue regeneration through secretion of various immunomodulatory factors.^[^ ^107^ ^]^ Gingival mesenchymal stem cells (GMSCs) encapsulated in alginate‐based photocrosslinkable hydrogels demonstrate excellent biocompatibility and osteogenic capacity in craniomaxillofacial bone repair.^[^ ^108^ ^]^

Hydrogel immunomodulation technology is advancing toward intelligent and multifunctional systems. Next‐generation smart responsive hydrogels can sense microenvironmental changes and respond accordingly, enabling precision disease treatment. Multifunctional composite hydrogels integrate mechanical support, immunomodulation, and tissue repair capabilities, providing comprehensive solutions for complex disease treatment.^[^ ^109^ ^]^

### 3.3 Scaffold

Scaffold‐based biomaterials play a crucial role in tissue engineering, particularly in bone tissue engineering, offering new approaches for bone defect repair that avoid permanent implants.^[^ ^110^, ^111^ ^]^ These materials achieve dual functions of tissue regeneration and immunomodulation through multi‐level design strategies (**Table** **2**). For example, scaffold materials can achieve tissue‐specific regeneration by loading growth factors. When bone morphogenetic proteins (BMP) such as BMP‐2 and BMP‐7 are incorporated into scaffolds, they specifically activate receptors on bone progenitor cells, triggering downstream signaling pathways that promote osteogenic differentiation.^[^ ^112^ ^]^

**Table 2:** Design strategies and immunomodulatory effects of different types of immunoregulatory scaffolds.

Scaffold classification	Specific materials/parameters	Immunomodulatory mechanism/effects	Reference
Growth factor‐loaded scaffolds	BMP‐2, BMP‐7 loaded scaffolds	Activate bone progenitor cell surface receptors, trigger osteogenic signaling pathways	[112]
Surface chemical modified scaffolds	Hydrophilic titanium surface	Inhibit TNF‐α, IL‐1α, IL‐1β and CCL2 expression	[113]
	Silk fibroin/IL‐4 modified PCL nanofiber scaffolds	Induce macrophage polarization toward M2 phenotype	[115]
Physical property‐regulated scaffolds			
Pore size control	Large pore scaffolds (≈360 μm)	Promote VEGF+ cell enrichment	[116]
Surface topography	Concave or convex scaffolds	Concave promotes osteogenic differentiation; convex inhibits cell proliferation	[117]
Material stiffness	PLLA/PLGA scaffolds	Stiff scaffolds support myotube formation; soft scaffolds cannot maintain myocyte viability	[118]
Material source classification	Biologically derived scaffolds	Activate Th2 immunity, upregulate IL‐4, Cd163, Mrc1	[120]
	Synthetic material scaffolds	Induce neutrophil infiltration	[120]
Degradable property scaffolds	Polylactic acid, polyglycolic acid	Degradation rate positively correlates with inflammation intensity, activates phagocytes	[121, 122]

Chemical modifications of scaffold surfaces directly influence their immunomodulatory capacity. Hydrophilic titanium surfaces, compared with micro‐rough surfaces, can significantly suppress the expression of pro‐inflammatory factors such as TNF‐α, IL‐1α, IL‐1β, and the chemokine (CC‐motif) ligand 2 (CCL2) in macrophages, demonstrating excellent anti‐inflammatory properties.^[^ ^113^ ^]^ The choice of terminal functional groups has a significant impact on immune responses: ‐CH_3_ groups trigger stronger inflammatory reactions than ‐COOH and ‐OH groups, manifested by increased recruitment of inflammatory cells.^[^ ^114^ ^]^ Polycaprolactone (PCL) nanofiber scaffolds modified with silk fibroin and IL‐4 successfully induce macrophage polarization toward the anti‐inflammatory M2 phenotype, achieving active immunomodulation.^[^ ^115^ ^]^

The physical properties of scaffolds have a decisive influence on cell behavior and tissue regeneration. Pore size directly regulates macrophage polarization: Large‐pore scaffolds (≈360 μm) promote VEGF+ cell enrichment and angiogenesis, facilitating the transition from M1 to M2 phenotype.^[^ ^116^ ^]^ Surface topography is equally critical: Concave scaffolds significantly enhance osteogenic differentiation of dental pulp stem cells, manifested by accelerated differentiation kinetics and thicker bone tissue formation, while convex scaffolds inhibit cell proliferation and matrix secretion.^[^ ^117^ ^]^ Material stiffness is a crucial parameter in regulating cell activity. In porous poly(L‐lactic acid) (PLLA)/poly(lactic‐co‐glycolic acid) (PLGA) scaffold systems, stiffer PLLA‐containing scaffolds support myotube formation, while pure PLGA soft scaffolds cannot maintain myocyte viability.^[^ ^118^ ^]^ Studies on polyacrylamide gel stiffness gradients further confirm that soft substrates induce neural differentiation of MSCs, medium stiffness promotes myogenic differentiation, while rigid substrates drive osteogenic differentiation.^[^ ^119^ ^]^

The source of scaffold materials influences the type of immune response they trigger. Biologically derived scaffolds primarily activate Th2‐mediated type II immune responses, upregulating anti‐inflammatory genes such as IL‐4, Cd163, and Mrc1, while synthetic material scaffolds tend to cause neutrophil infiltration, with these differences closely related to material stiffness and dimensions.^[^ ^120^ ^]^

The impact of material degradation processes on immune responses cannot be overlooked. The degradation rate of biodegradable polymers such as polylactic acid and polyglycolic acid correlates positively with inflammation intensity, and their degradation products can activate phagocytes and induce superoxide anion production.^[^ ^121^, ^122^ ^]^ This finding emphasizes the importance of controlling degradation kinetics to optimize material biocompatibility.

Scaffold material design is advancing toward multifunctional integration. Ideal scaffolds should provide structural support while achieving precise regulation of the immune microenvironment through surface modification, physical structure optimization, and degradation control. In‐depth understanding of the molecular mechanisms underlying material‐immune system interactions will lay the foundation for developing next‐generation smart responsive scaffold materials, ultimately enabling clinical translation in tissue engineering and regenerative medicine.

## 4 Common Chronic Inflammatory Diseases and Material Immune Regulation Therapy

**Figure 4:**
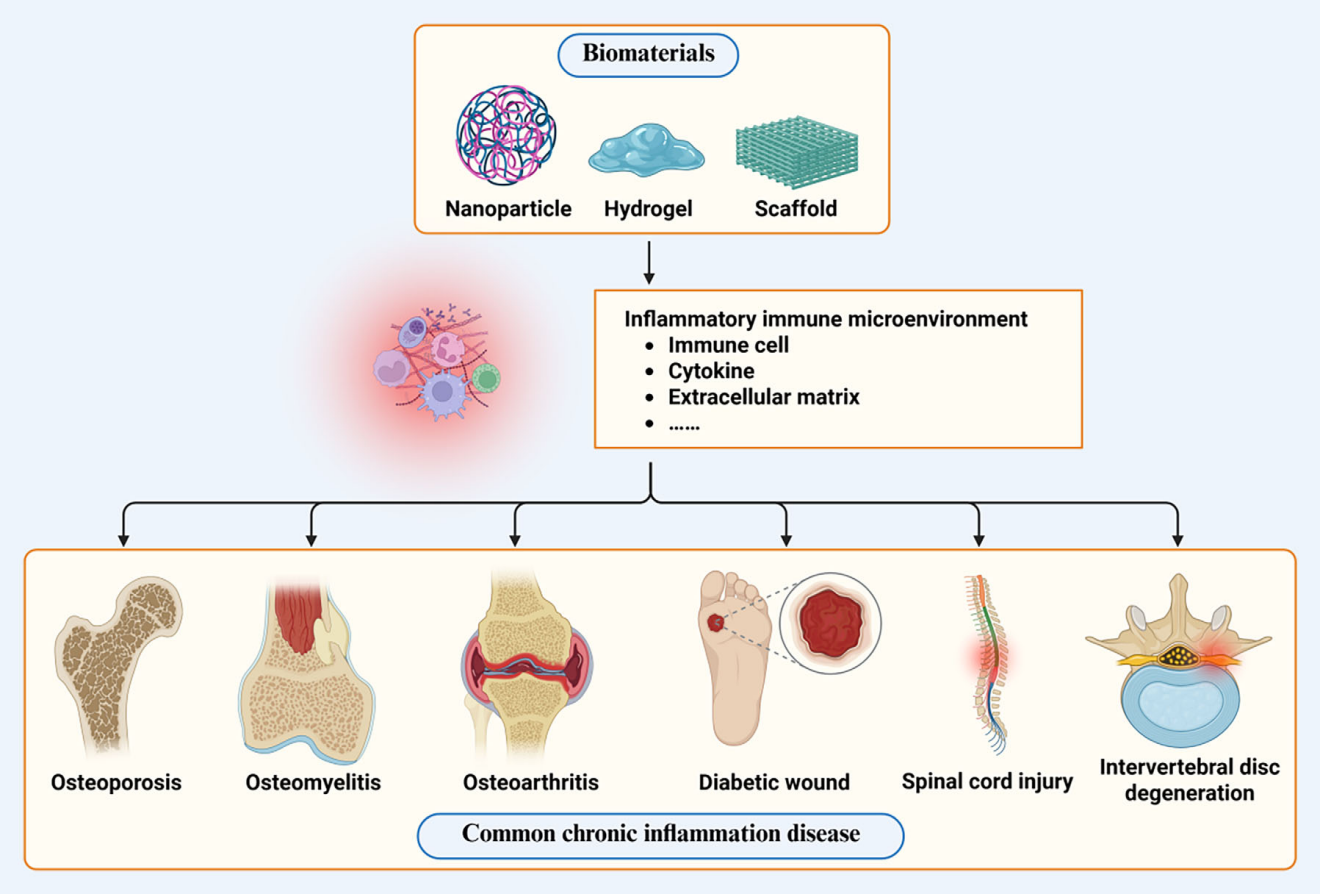
Common chronic inflammatory diseases of bone and soft tissue and immune regulation therapy of biomaterials. Osteoporosis is a disease of bone metabolism mediated by immune microenvironment imbalance. Osteomyelitis is a bone tissue infection characterized by biofilm‐mediated immunosuppression. Osteoarthritis (OA) is a chronic inflammatory disease characterized by polarization of M1 macrophages, infiltration of T cells, and release of pro‐inflammatory factors such as MMPs. Diabetic wound is a chronic inflammation characterized by high reactive oxygen species (ROS) levels and immune imbalance. Spinal cord injury (SCI) is a chronic disease caused by a cascade of multiple systems such as injury, inflammation, ischemia and oxidative stress. Intervertebral disc degeneration (IVDD) is a pathological process in which the structure of the intervertebral disc induces autoimmune response and persistent inflammation. Biomaterials optimize the treatment of common chronic inflammatory diseases by regulating the components of the immune microenvironment, such as immune cells, cytokines, and extracellular matrix. Created in https://BioRender.com.

The pathophysiological features of chronic inflammatory diseases are primarily characterized by persistent low‐grade inflammation, immune cell dysfunction, imbalance between pro‐inflammatory and anti‐inflammatory factors, and disruption of tissue microenvironment homeostasis. Although these diseases occur in different tissues and organs, they share similar immunopathological mechanisms, such as imbalanced M1/M2 macrophage polarization, excessive neutrophil activation, abnormal T cell subset distribution, and cytokine network dysfunction. This chronic inflammatory microenvironment not only impedes normal tissue repair processes but also leads to secondary tissue damage and functional impairment. Immunomodulatory biomaterials, through their unique physicochemical properties and biological activities, provide innovative therapeutic strategies for these diseases (**Figure** **4**).

### 4.1 Osteoporosis

#### 4.1.1 The Immune Microenvironment of Osteoporosis

Osteoporosis is a systemic bone disease characterized by low bone mass and microarchitectural impairment of bone tissue, with a consequent increase in bone fragility and susceptibility to fracture.^[^ ^123^ ^]^ Postmenopausal osteoporosis and senile osteoporosis are the main pathophysiological processes associated with significant bone loss.^[^ ^124^ ^]^ The mechanisms of osteoporosis are continuously being explored, and it is recognized that the immune system plays an important role in the development of osteoporosis. Bone homeostasis requires continuous bone resorption and constant remodeling to adapt to loading and replace damaged bone tissue. The coordinated balance between bone resorption by osteoclasts (OC) and bone formation by osteoblasts (OB) maintains a relatively stable bone mass during adulthood. However, imbalance in bone remodeling with increased resorption compared with formation may lead to bone loss and even osteoporosis.^[^ ^124^, ^125^ ^]^ These processes require the involvement of immune responses. Considering the close relationship between the immune system and the skeletal system, osteoporosis is accompanied by changes in the immune microenvironment.^[^ ^126^ ^]^ Treatment by targeting bone and immunity offers additional therapeutic options and research directions for osteoporosis.

Chronic inflammation is an important factor in the development of osteoporosis.^[^ ^127^ ^]^ The polarization states of macrophages switch to each other in response to changes in the microenvironment. M1/M2 ratio increases in osteoporosis.^[^ ^128^ ^]^ Accumulation of pro‐inflammatory cytokines produced by M1 gradually leads to enhanced OC activity in osteoporosis.^[^ ^129^ ^]^ In addition to secreting cytokines, M1 macrophages themselves have the potential to differentiate into OCs and can inhibit the secretion of osteoprotegerin (OPG).^[^ ^130^ ^]^ Their role in enhancing OC differentiation needs to be further investigated. In contrast, M2 macrophages secrete anti‐inflammatory molecules such as CCL18, CCL22, IL‐10, and osteogenic cytokines including bone morphogenetic protein 2 (BMP‐2), TGF‐β, VEGF, and osteopontin, tending to inhibit bone resorption and promote osteogenesis.^[^ ^131^, ^132^ ^]^ Thus, regulation of cytokines in the microenvironment in favor of M2 macrophages may be an effective therapeutic strategy for improving osteoporosis.

T lymphocytes play essential roles in osteoporosis. Studies have found that Th17 cells are increasingly present in the bone marrow of mice after ovariectomy (OVX).^[^ ^133^ ^]^ Th17 cells regulate bone mass via two main mechanisms: 1) They highly express receptor activator of NF‐κB ligand (RANKL), which binds to RANK on osteoclast precursors to promote their differentiation into osteoclasts, and 2) IL‐17 produced by Th17 cells stimulates osteoclast‐supporting cells, such as synovial fibroblasts, as well as osteoclasts themselves, to upregulate RANKL expression, thereby enhancing bone resorption.^[^ ^57^ ^]^

Estrogen deficiency stimulates B lymphocyte production in the bone marrow.^[^ ^134^ ^]^ Under physiological conditions, B cells express both osteoprotegerin (OPG) and RANKL. B cells account for more than 60% of total bone marrow OPG production,^[^ ^135^ ^]^ while, in osteoporosis, the inflammatory microenvironment stimulates B cells to produce larger amounts of RANKL, resulting in an increased RANKL/OPG ratio, which drives disease progression.^[^ ^136^ ^]^ This conclusion is supported by the studies that ovariectomy in mice increased the number of RANKL‐expressing B lymphocytes in the bone marrow and that mice lacking RANKL in their B lymphocytes were partially protected from ovariectomy‐induced bone loss in cancellous bone.^[^ ^137^, ^138^ ^]^

Neutrophils are involved in inflammation‐induced osteoporosis.^[^ ^139^ ^]^ Studies have shown that OVX mice exhibited increased neutrophil infiltration in inflammation and might therefore be involved in osteoporosis progression.^[^ ^140^ ^]^ Neutrophils can produce chemokines such as CCL2 and CCL20 to recruit Th17 cells, leading to bone loss.^[^ ^141^ ^]^ In addition, neutrophils express RANKL and increased expression of RANKL is closely associated with inflammation.^[^ ^142^ ^]^

Dendritic cells (DCs) also contribute to inflammation‐mediated OC formation. Estrogen regulates the number of DCs expressing IL‐7 and IL‐15. Studies have shown that in OVX mice with osteoporosis, DCs maintained for a long time and expressed more IL‐7 and IL‐15 in the absence of estrogen, producing cytokines that drive inflammation‐mediated bone loss.^[^ ^143^ ^]^ A more direct effect is demonstrated by the fact that in the presence of RANKL and M‐CSF, DCs can *trans*‐differentiate into OCs, newly formed OCs can summon more DCs by inducing DC tropism, and thus forming an OC‐DC loop to continue increasing bone destruction.^[^ ^144^, ^145^ ^]^

Cytokines secreted by immune cells play a crucial role in the development of osteoporosis. Pro‐osteogenic factors BMP‐2 and IL‐4 are significantly reduced in patients with senile osteoporosis and postmenopausal osteoporosis, where BMP‐2 stimulates osteogenesis and bone mineralization,^[^ ^146^, ^147^, ^148^ ^]^ and IL‐4 inhibits osteoclast function while promoting osteoblast maturation.^[^ ^149^, ^150^, ^151^ ^]^ In contrast, pro‐resorptive factors TNF‐α and IL‐6 are significantly elevated in osteoporosis patients. TNF‐α inhibits osteoblast activity while stimulating osteoclast differentiation, playing a particularly prominent role in postmenopausal osteoporosis.^[^ ^152^, ^153^, ^154^ ^]^ IL‐6, as a bone resorption‐promoting factor, shows increased expression in postmenopausal and osteoporosis patients, exacerbating bone loss.^[^ ^155^ ^]^

#### 4.1.2 Current Treatments and the Immunomodulatory Effects of Biomaterials

Current standard treatment for osteoporosis mainly comprises lifestyle interventions and pharmacotherapy. Common antiresorptive agents include bisphosphonates,^[^ ^156^ ^]^ selective estrogen receptor modulators (e.g., raloxifene),^[^ ^157^ ^]^ and anti‐RANKL monoclonal antibodies (e.g., denosumab),^[^ ^158^ ^]^ while anabolic therapy is represented by recombinant human parathyroid hormone (PTH1‐34). For patients with severe osteoporosis or concomitant fractures, surgical reinforcement or implant‐based repair are often required. These treatments can substantially reduce fracture risk and improve bone mineral density, but several limitations remain: Long‐term bisphosphonate use may increase the risk of osteonecrosis of the jaw and atypical femoral fractures;^[^ ^159^ ^]^ many antiresorptive therapies do not simultaneously promote bone formation, resulting in limited efficacy and potential metabolic rebound after discontinuation. Moreover, current approaches predominantly focus on inhibiting bone resorption or promoting bone formation, with insufficient regulation of immune microenvironment imbalance and inflammatory mediator‐driven bone loss mechanisms, resulting in limited efficacy in inflammation‐related or senile osteoporosis and a lack of individualized treatment strategies. Given that immune cells, via secretion of diverse cytokines, play complex and critical roles in maintaining bone metabolic homeostasis, targeting and modulating the immune microenvironment in osteoporosis may represent a promising complementary strategy to fill existing therapeutic gaps and enhance clinical efficacy.

**Figure 5:**
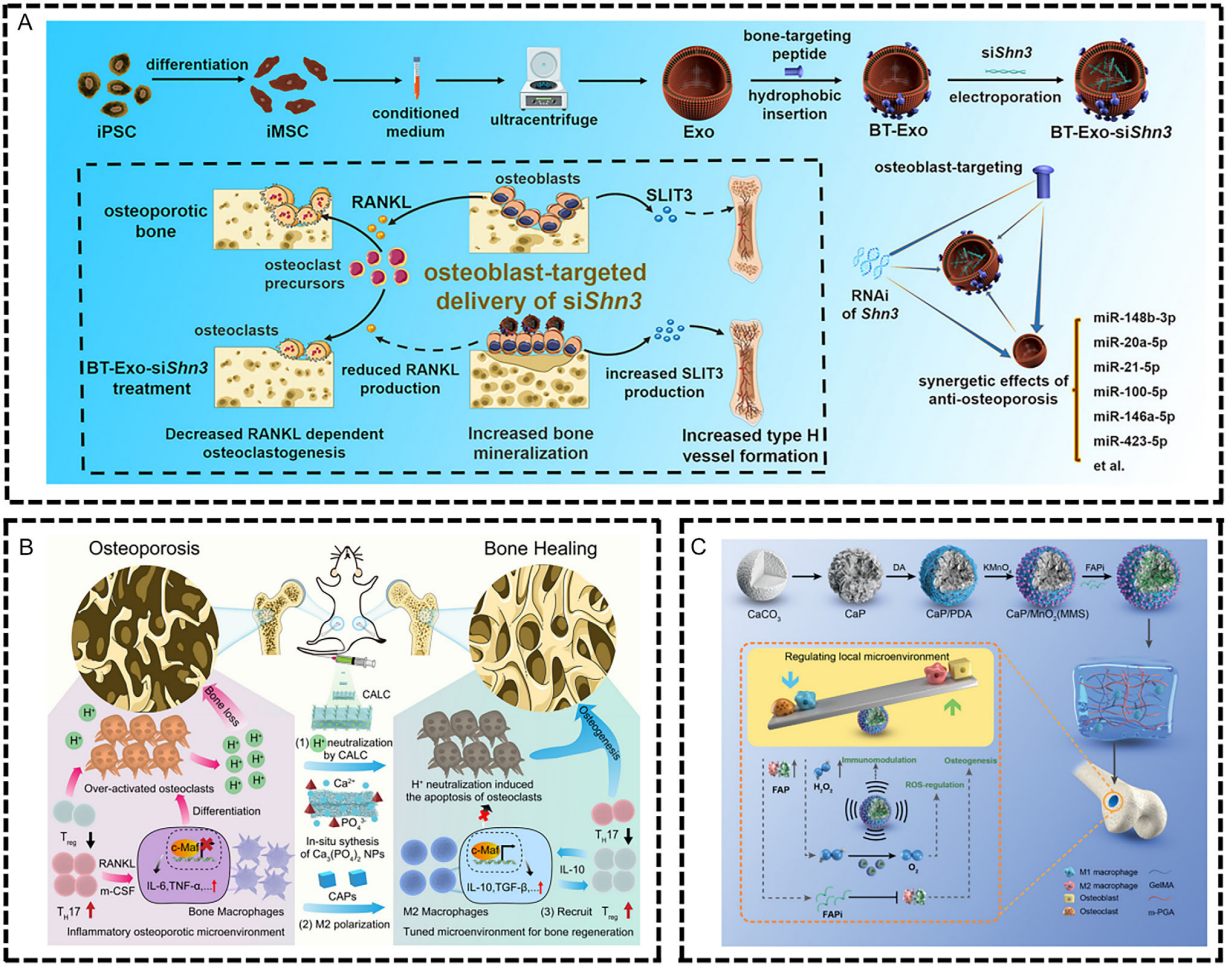
Biomaterials for osteoporosis treatment. A) Bone targeting peptide modified payload exosomes (BT‐Exo‐siShn3) target Shn3 gene siRNA delivery to osteoblasts to treat osteoporosis. The silencing of Shn3 gene in osteoblasts enhanced osteogenic differentiation and decreased autologous RANKL expression, thus inhibiting osteoclast formation. In addition, Shn3 gene silencing increases the production of SLIT3, which promotes blood vessel formation. Reproduced with permission.^[^ ^163^ ^]^ Copyright 2022, Elsevier. B) Acid neutralization and immune regulation of calcium‐aluminum layer double hydroxide (CALC) reverse osteoporosis. CALC nanosheets can neutralize the acidic microenvironment of osteoporosis, and Ca^2+^ released by degradation of LDH reacts with endogenous phosphate to generate calcium phosphate nanoparticles (CAPs), resulting in the phenotype anti‐inflammatory differentiation of bone macrophages M2 through c‐Maf transcription factor pathway, the enhancement of Treg cell activity, and the deactivation of T helper 17 cells (Th17). Reproduced with permission.^[^ ^167^ ^]^ Copyright 2022, American Chemical Society. C) Composite hydrogels containing MnO_2_. Coated CaP microspheres containing FAPi are used to repair osteoporotic bone defects, and MnO_2_ eliminates ROS, thereby generating oxygen in the microenvironment. CaP microspheres degrade slowly and continuously release FAPi to inhibit FAP, thereby regulating immune response and bone formation. Reproduced with permission.^[^ ^168^ ^]^ Copyright 2023, Wiley‐VCH GmbH.

Nanomaterial‐based drug delivery systems have demonstrated tremendous potential in the treatment of osteoporosis animal models. Nanoparticles (NPs), due to their size similarity to bone tissue structure and high surface area‐to‐volume ratio, facilitate the adsorption and bioactivity of adjacent proteins and cells, making them effective drug delivery platforms.^[^ ^160^ ^]^ Poly(lactic‐co‐glycolic acid) (PLGA) nanoparticles as a delivery system can achieve local delivery of BMP‐2 at lesion sites and can be used for spatially and temporally controlled growth factor delivery.^[^ ^161^ ^]^ In ovariectomized rat osteoporosis models, parathyroid hormone‐loaded and polyethylene glycol‐modified chitosan nanoparticles administered via oral route prolonged drug half‐life, achieved sustained drug release, and showed increased bone mineralization in in vitro osteoblast cultures.^[^ ^162^ ^]^ Nanoparticles also demonstrate advantages in gene therapy. CH6 aptamer‐functionalized lipid nanoparticles achieved targeted delivery of osteogenic siRNA in rat models, while bone‐targeting peptide‐modified loaded exosomes successfully delivered Shn3 gene siRNA to osteoblasts in in vitro cell experiments (**Figure** **5**A).^[^ ^163^, ^164^ ^]^ Iron oxide nanoparticles possess unique magnetic properties and good biocompatibility, promoting mesenchymal stem cell osteogenic differentiation and inhibiting osteoclast formation in in vitro experiments. When combined with static magnetic fields, they synergistically enhance effects on osteoblast and osteoclast differentiation and also exhibit anti‐inflammatory effects in macrophage cells.^[^ ^165^, ^166^ ^]^

Combining immunomodulatory substances with biomaterials represents a highly promising therapeutic strategy. Fu et al. designed a nanocatalytic drug, calcium–aluminum layered double hydroxide (CALC) loaded with calcein. In ovariectomy‐induced osteoporotic mouse models, CALC nanosheets neutralize the acidic microenvironment at osteoporotic lesion sites and reduce osteoclast activity, while inducing macrophage M2 phenotype differentiation in in vitro RAW264.7 macrophage experiments. In vivo experiments demonstrate that this system enhances regulatory T cell (Treg) activity and inactivates Th17 cells, effectively reversing the inflammatory microenvironment (Figure 5B).^[^ ^167^ ^]^ Chen et al. dispersed fibroblast activation protein inhibitor‐loaded MnO_2_‐coated calcium phosphate microspheres (FAPi‐MMS) in methacrylated PGA/methacrylated gelatin (m‐PGA/GelMA) hydrogel to prepare a multifunctional composite hydrogel.^[^ ^168^ ^]^ In osteoporotic rat femoral defect models, this composite hydrogel improves the oxidative stress microenvironment by scavenging reactive oxygen species (ROS). In vitro macrophage experiments confirm its ability to enhance macrophage M2 polarization and inhibit M1 polarization, thereby promoting bone defect repair. However, the addition of MMS alters the microstructure and mechanical properties of the composite hydrogel, which may affect the mechanosensing and cell differentiation of bone marrow mesenchymal stem cells (Figure 5C).^[^ ^168^ ^]^

Biomaterials with bone‐enhancing and antifracture properties are widely applied in osteoporosis treatment.^[^ ^169^ ^]^ Wang et al. develop a functional coating by forming strontium‐doped nanostructures on titanium surfaces and introducing multilayered chrysin‐loaded silk fibroin nanoparticles to modify the titanium substrate.^[^ ^170^ ^]^ In ovariectomized rat femoral fracture models, controlled‐release chrysin effectively regulates macrophage polarization from M1 to M2 phenotype in in vitro RAW264.7 macrophage experiments, promoting osteoblast differentiation through paracrine signaling. Simultaneously, the sustained release of Sr ions from the coating further promotes osteogenesis in in vitro osteoblast cultures and enhances new bone formation around implanted tissue in vivo, demonstrating strong anti‐inflammatory and osteogenic capabilities.^[^ ^170^ ^]^

Recombinant human parathyroid hormone (PTH1‐34) is approved for clinical anti‐osteoporosis treatment due to its excellent osteogenic activity, but it also has catabolic effects that promote osteoclast formation.^[^ ^171^ ^]^ Yi Wang et al. develop a novel PTH‐related peptide‐1 (PTHrP‐1), which regulates M1 macrophage polarization and reduces osteoclast activity in in vitro bone marrow macrophage and osteoclast precursor cell experiments, while retaining the osteogenic and pro‐angiogenic properties of PTH. Through PTHrP‐1‐functionalized multifunctional calcium phosphate ceramic scaffolds in rabbit femoral defect models, the system achieves sustained release of PTHrP‐1 and improves the osteogenic microenvironment.^[^ ^172^ ^]^

Bone marrow mesenchymal stem cells (BMSCs) possess multidirectional differentiation potential, bone marrow supporting capacity, anti‐inflammatory, and immunomodulatory properties, making them multifunctional therapeutic cells.^[^ ^173^ ^]^ Bai et al. combine rigid 3D‐printed porous metal scaffolds with flexible supramolecular polysaccharide hydrogels, encapsulating BMSCs and BMP‐2.^[^ ^174^ ^]^ In osteoporotic rabbit femoral defect models, BMSCs within the scaffolds maintain good cell viability, BMP‐2 promotes BMSC osteogenic differentiation in in vitro experiments, and the osteoporotic condition of bone surrounding the bioactive interface is significantly improved.^[^ ^174^ ^]^

Immunomodulatory biomaterials‐mediated immune responses in bone repair demonstrate excellent anti‐osteoporotic capacity and repair efficacy while regulating the immune microenvironment. The experimental results of these materials further suggest that combined immunomodulatory strategies may be a promising approach for promoting the repair of osteoporotic bone defect.

### 4.2 Osteomyelitis

#### 4.2.1 The Immune Microenvironment of Osteomyelitis

Osteomyelitis is an inflammatory disease of bone and bone marrow caused by pathogenic microorganisms, characterized by bone tissue destruction.^[^ ^175^ ^]^ *Staphylococcus aureus* (*S. aureus*) is the most common causative pathogen, which can infect bone tissue through contiguous spread from adjacent tissues, direct trauma, or hematogenous dissemination.^[^ ^176^ ^]^ Compared with other pathogens, *S. aureus* possesses unique capabilities for bone tissue invasion, colonization, and proliferation.^[^ ^177^ ^]^ This bacterium can reach the bone surface through iatrogenic procedures or hematogenous routes, and readily adheres to soft tissues, bone, or metallic implants.^[^ ^178^ ^]^

Bacterial invasion typically triggers a robust inflammatory response, including innate immune responses mediated by macrophages and neutrophils, as well as adaptive immune responses mediated by T cells, B cells, and pathogen‐specific antibodies.^[^ ^179^ ^]^ These immune cells release abundant inflammatory factors (such as TNF‐α, IL‐1, IL‐6, and IL‐17) to eliminate pathogens. However, studies have found that macrophages not only fail to effectively kill phagocytosed *S. aureus* but also induce multiple antibiotic resistance,^[^ ^180^ ^]^ leading to the transition to chronic and refractory osteomyelitis.

As infection persists and becomes chronic, *S. aureus* tends to form biofilm phenotypes. Biofilm formation not only reduces the bactericidal activity of neutrophils, but the bacteria also secrete toxins that disrupt immune cell membranes causing lysis while inducing macrophages at chronic infection sites to polarize toward the pro‐healing M2 phenotype.^[^ ^181^ ^]^ Additionally, *S. aureus* can regulate the expression of myeloid‐derived suppressor cells, inhibiting pro‐inflammatory cytokine production and T cell recruitment to infection sites, thereby maintaining bacterial survival.^[^ ^182^, ^183^ ^]^

T and B cell responses are crucial for host defense against bone infections, but *S. aureus* can interfere with adaptive immunity through multiple mechanisms. In animal osteomyelitis models, bacterial biofilms skew CD4+ helper T cells toward Th1 and Th17 responses, resulting in ineffective intracellular pathogen clearance.^[^ ^184^, ^185^ ^]^ The proportion of regulatory T cells (Treg) is elevated in the blood of chronic osteomyelitis patients, while T cell proliferation is reduced in infected bone tissue,^[^ ^186^, ^187^ ^]^ which may be due to Treg cell‐specific suppression of T cell‐mediated immune responses. *S. aureus* super antigens can cause antigen‐independent T cell activation, primarily releasing Th1 cytokines (IL‐2, IFN‐γ, and TNF‐β), while Th2 cytokine (IL‐4 and IL‐5) responses are weak.^[^ ^188^ ^]^ The lack of Th2 response may inhibit neutralizing antibody production and impair extracellular bacterial clearance.^[^ ^189^, ^190^ ^]^

Regarding humoral immunity, *S. aureus* can block antibody‐mediated phagocytosis while inducing proliferative apoptosis of B cells, thereby regulating B cell survival and function.^[^ ^191^, ^192^ ^]^ Particularly noteworthy is that direct infection of osteoblasts by *S. aureus* has significant pathogenic importance, as it can induce the secretion of pro‐osteoclastogenic factors, leading to pathological bone loss.^[^ ^193^, ^194^ ^]^ These complex immune evasion mechanisms work synergistically, making *S. aureus* osteomyelitis a major clinical therapeutic challenge.

#### 4.2.2 Current Treatments and the Immunomodulatory Effects of Biomaterials

The standard treatment for osteomyelitis primarily involves a combination of surgical intervention and antibiotics: Acute or chronic osteomyelitis often requires thorough surgical debridement, removal of necrotic tissue, and local or systemic antibiotic therapy based on the pathogen, sometimes necessitating implantation of antibiotic bone cement or biodegradable carriers to maintain high local drug concentrations; for implant‐associated infections, removal and staged reconstruction are often considered.^[^ ^195^ ^]^ Despite these strategies being clinical cornerstones, they face multiple limitations: Bacterial biofilms and intracellular latent infections make it difficult for systemic antibiotics to completely eradicate pathogens, leading to frequent recurrent infections and drug resistance issues; broad‐spectrum or long‐term medication causes systemic toxic side effects and hepatorenal burden; surgical trauma is extensive with slow recovery and potential functional impairment; local drug delivery carriers may become foreign bodies and hinder tissue integration; furthermore, infection sites are often accompanied by an immunosuppressive microenvironment, and antimicrobial therapy alone cannot restore impaired immune clearance function, resulting in high recurrence rates and prolonged treatment cycles.^[^ ^196^ ^]^ Although perioperative systemic antibiotics help prevent infection, their fluctuating blood concentrations and the need for repeated dosing limit their effectiveness.^[^ ^197^ ^]^ By contrast, achieving sustained high‐concentration local delivery at the infected site can more effectively penetrate bone tissue, enhance local antimicrobial efficacy, and reduce systemic toxicity.^[^ ^198^ ^]^ Consequently, local antibiotic delivery using biomaterial carriers has become a prominent and promising approach in current research and clinical practice.

Traditionally, antibiotic delivery systems are based on the acrylic material poly‐methylmethacrylate (PMMA) in the form of cements or beads. These can be used in combination with many antibiotics and have been widely used in surgical procedures. Whereas the significant disadvantage is that they can act as foci of infection, this disadvantage can be overcome by the use of biodegradable antimicrobial products.^[^ ^199^ ^]^ The use of active coatings on implants releases preadult rated bactericidal agents such as antibiotics, antiseptics, metal ions, nonmetallic components and functional peptides, thereby reducing infection.^[^ ^200^ ^]^ Silver‐coated and gentamicin‐coated prostheses have been used as giant prostheses for the fixation of large periarticular bone defects and fractures.^[^ ^201^, ^202^ ^]^ Liao et al. made a multilayer film structure coatings from montmorillonite with poly‐L‐lysine and chlorhexidine to achieve active release of antimicrobial agents with progressive degradation of the multilayer structure by bacteria enzymes to achieve bactericidal effect.^[^ ^203^ ^]^ Unfortunately, there are no effective antimicrobial coatings that are bacterial anti‐adhesion substrate‐independent or have long‐term biofilm inhibition.

**Figure 6:**
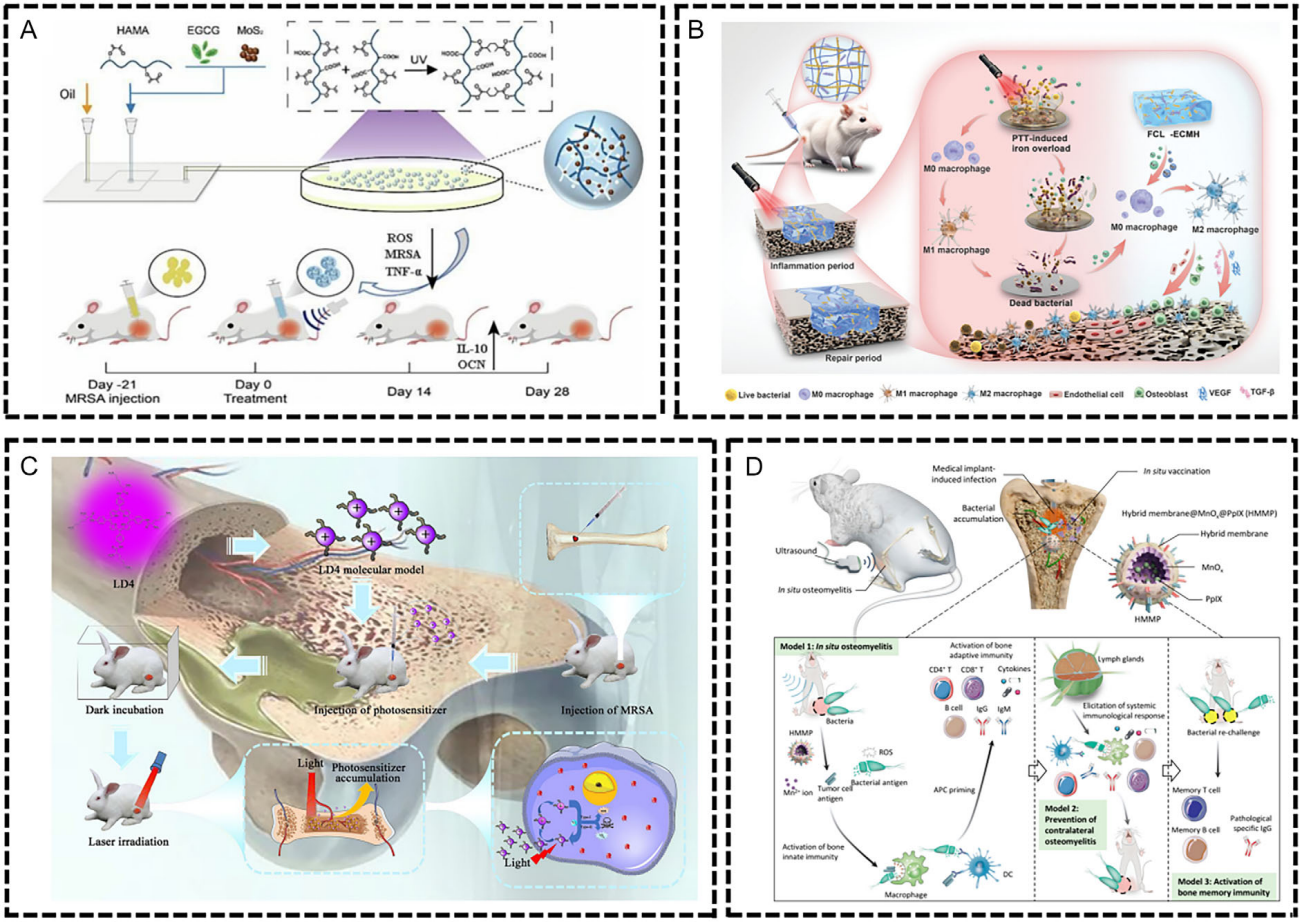
Biomaterials for the treatment of osteomyelitis. A) The manufacture of injectable EMgel and in situ injection of EMgel into SD rats with chronic osteomyelitis infected with MRSA to kill bacteria and promote bone regeneration. Reproduced with permission.^[^ ^208^ ^]^ Copyright 2024, Elsevier. B) FCL‐ECMH induces the regeneration of infected tissues by regulating the polarization of macrophages during the healing process. Reproduced with permission.^[^ ^214^ ^]^ Copyright 2024, Wiley‐VCH GmbH. C) Osteomyelitis was treated with photodynamic antibacterial chemotherapy combining the novel photosensitizer LD4 and antibiotics. Reproduced with permission.^[^ ^215^ ^]^ Copyright 2022, Frontiers. D) Biological simulation nanomedicine HMMP is used for in situ vaccination in osteomyelitis models. HMMP enables rapid antigen release, activates antigen‐presenting cells, and thereby triggers cellular and humoral immune responses. Reproducedwith permission.^[^ ^217^ ^]^ Copyright 2022, American Chemical Society.

Nanosized materials with unique physical and chemical properties, potent bactericidal activity and specific mechanisms show great potential to eradicate resident bacteria and pathogenic biofilms.^[^ ^204^ ^]^ Synthetic biocompatible materials such as PLGA, polyanhydride, polycaprolactone, bioactive glass, scaffolds, and natural polymers such as collagen, hyaluronic acid carboxymethyl cellulose, and chitosan or metal nanoparticles are used as platforms to act as carriers for antibiotic or drug combination loading. Nanomaterials can be designed to be activated in response to unique biofilm pathological microenvironments to accurately carry, retain and release drugs. For example, mesoporous bioactive glass (MBG) and PLGA composite scaffolds offer controlled degradability, improved hydrophilicity, sustained antibiotic release efficiency, and reliable osteoinductivity.^[^ ^205^ ^]^ Vancomycin‐loaded MBG/PLGA composite scaffolds prolonged effective drug release by up to 8 weeks, and the rate of scaffold degradation matched the rate of new bone formation.^[^ ^205^ ^]^ A combined polymeric formulation of a mixture of polyanhydride and polylactide with ofloxacin inhibited the growth of *S. aureus*, *E. coli*, and *P. aeruginosa* up to 89 days after treatment.^[^ ^206^ ^]^ Teicoplanin‐loaded lipid liquid‐crystalline made by Chen et al. delivered teicoplanin sustained release for 36 days compared to 48 h for the control gel.^[^ ^207^ ^]^ Guo et al. prepared an acoustically responsive multifunctional hydrogel microsphere‐bomb (EM gel), which encapsulated natural polyphenol epigallocatechin‐3‐gallate (EGCG) and bioactive MoS2. It showed a lower inflammatory response and higher osteocalcin (OCN) expression in the ultrasonic treatment group (**Figure** **6**A).^[^ ^208^ ^]^ Shahnaz Qadri et al. found that silver‐copper‐boron composite nanoparticles prolonged the release of their component ions (Ag, Cu), which could replace the use of antibiotics, and 99% of bacteria were cleared in an induced osteomyelitis mouse model.^[^ ^209^ ^]^ Despite the various advantages of nanoparticles over their parent materials, their small size produces various toxicities compared to larger particles.^[^ ^210^ ^]^ Studies on the inhibitory effect of nanoparticles on persistent bacteria in biofilms are still scarce.

Targeting biofilms, new anti‐biofilm mechanisms such as photothermal therapy (PTT) and photodynamic therapy (PDT) with smart nanoparticles might be a more promising way to kill persistent cells.^[^ ^211^ ^]^ PTT relies on photothermal agents to convert light into local heat, which disrupts the integrity of bacteria or the structure of biofilms through local thermotherapy and can also facilitate the diffusion of photosensitizers into biofilms. PDT, meanwhile, is performed in the near‐infrared or visible light irradiation to stimulate ROS and disrupt the integrity of bacterial cell membranes.^[^ ^212^, ^213^ ^]^ Lu et al. designed a novel composite hydrogel, FCL‐ECMH, which encapsulates the vermiculite functional core layer (FCL) in an acellular extracellular matrix hydrogel (ECMH). During PTT, it demonstrated high efficiency in converting light into heat, promoting bacterial ferroptosis‐like death and regulating macrophage polarization, as well as enhancing antibacterial and tissue repair capabilities (Figure 6B).^[^ ^214^ ^]^ Yin et al. developed an amino tetraphenylporphyrin compound modified by basic amino acids called LD4, which has a broader photoinactivation range than other photosensitizers and can effectively kill gram‐positive and gram‐negative bacteria. Photosensitizer LD4 combined with antibiotics in photodynamic antimicrobial chemotherapy is effective for *S. aureus*‐induced osteomyelitis (Figure 6C).^[^ ^215^ ^]^

In addition, bacteriophages have been investigated as a therapeutic strategy and used in several clinical studies demonstrating their safety and possible efficacy. Phage‐derived lysins are also frequently used independently or in combination with antibiotics. For example, in a murine model of implant‐associated osteomyelitis, a combination of systemic and topically applied phage‐derived lysin improved eradication of *S. aureus* infection after debridement and implant retention.^[^ ^216^ ^]^

A novel therapeutic strategy targeting the immune system is the use of vaccines. Han Lin et al. presented an in situ bacterial vaccination strategy using a hybrid nanovesicle as a platform dependent on bacteria‐associated antigens (BAA) and immune‐activating tumor antigens. This approach effectively activates innate and adaptive immune responses against bacteria‐derived acute osteomyelitis and its later recurrence. Systemic immune response was successfully triggered, and long‐lived B cell and T cell immunity was also triggered to prevent recurrent infections (Figure 6D).^[^ ^217^ ^]^

However, there are still obstacles to designing bacterial vaccines. Vaccines deliver one or several antigens and adjuvants to the antigen‐presenting cells (APCs) to trigger antigen‐specific cytotoxic T lymphocyte (CTL) and humoral responses, but their use in the treatment of antimicrobial infections has mostly failed due to highly mutated antigenic epitopes.^[^ ^218^ ^]^ Vaccines specifically targeting the biofilm matrix components present in biofilm growth offer possible directions.^[^ ^219^ ^]^ Reversal of the immunosuppressive microenvironment of osteomyelitis to an immune‐infiltrating microenvironment remains a major challenge in the treatment of osteomyelitis, but also remains a promising therapeutic avenue.

### 4.3 OA

#### 4.3.1 The Immune Microenvironment of OA

Osteoarthritis (OA) is a degenerative disease characterized by synovial inflammation, cartilage loss, subchondral bone changes, and osteophyte formation. Although traditionally considered a “wear and tear” disease, increasing evidence indicates that low‐grade inflammation plays a key role in OA pathogenesis, suggesting that the immune microenvironment plays an important role in the occurrence and progression of OA.^[^ ^220^ ^]^

In the immune response of OA, innate immune cells play crucial roles. In early inflammation, neutrophils are the first to be recruited to the injury site, exerting chemotactic and phagocytic functions by secreting pro‐inflammatory mediators and elastase.^[^ ^221^ ^]^ Macrophages are activated and polarized under stimulation by pathogen‐associated molecular patterns (PAMPs), damage‐associated molecular patterns (DAMPs), and inflammasomes.^[^ ^222^ ^]^ M1‐type macrophages secrete pro‐inflammatory mediators and matrix metalloproteinases (MMPs) under IFN‐γ and lipopolysaccharide stimulation, promoting cartilage degradation and inhibiting chondrogenic differentiation of stem cells; whereas M2‐type macrophages secrete anti‐inflammatory factors and pro‐chondrogenic factors, facilitating cartilage repair.^[^ ^223^ ^]^

Adaptive immunity is equally important in OA. T cells are the main component of synovial infiltration in OA patients, including CD4+ and CD8+ T cells.^[^ ^224^ ^]^ T cell numbers are significantly elevated in the synovial fluid of OA patients, and these cells promote extracellular matrix (ECM) degradation and remodeling by secreting cytokines and growth factors.^[^ ^225^, ^226^ ^]^ CD4+ T cells can differentiate into different subtypes and exert distinct functions: Th1 cells are elevated in the synovium;^[^ ^224^ ^]^ Th2 cells secrete anti‐inflammatory factors such as IL‐4 and IL‐5;^[^ ^227^ ^]^ Th17 cells release IL‐17, inhibiting proteoglycan synthesis and enhancing proteolytic enzyme activity, thereby promoting cartilage degradation;^[^ ^223^ ^]^ Treg cells maintain immune homeostasis by secreting anti‐inflammatory molecules such as IL‐10 and TGF‐β while inhibiting IL‐6 production.^[^ ^228^, ^229^ ^]^ B cells and dendritic cells (DCs) have dual roles in OA. B cells can both impair cartilage repair by secreting cytokines and antibodies, and regulate autoimmune responses to promote inflammation resolution.^[^ ^230^ ^]^ Mature DCs inhibit chondrogenic differentiation of mesenchymal stem cells by secreting pro‐inflammatory factors, while immature DCs have immunoregulatory effects and promote chondrogenic differentiation.^[^ ^231^, ^232^ ^]^

The cytokine network plays a central role in regulating the OA immune microenvironment. Pro‐inflammatory factors such as IL‐1β, IL‐6, IL‐17, and TNF‐α lead to cartilage destruction by inhibiting synthesis or promoting degradation of matrix components including type II collagen, proteoglycans, and glycosaminoglycans.^[^ ^223^ ^]^ Oxidative stress caused by reactive oxygen species (ROS) can promote chondrocyte senescence and apoptosis, and facilitate matrix degradation by altering collagen and protein synthesis.^[^ ^233^ ^]^ Conversely, anti‐inflammatory factors such as IL‐4 can protect cartilage from MMP‐induced damage, and IL‐10 promotes Col 2 and GAG expression, favoring cartilage formation.^[^ ^234^, ^235^ ^]^ These inflammatory factors play key roles in OA progression by activating signaling pathways such as NF‐κB, regulating the expression of matrix‐degrading proteins in chondrocytes.^[^ ^236^ ^]^

#### 4.3.2 Current Treatments and the Immunomodulatory Effects of Biomaterials

Conventional treatment for osteoarthritis primarily involves conservative management, including lifestyle modifications, analgesic and anti‐inflammatory medications, local injection therapies, and surgical interventions when conservative treatment fails.^[^ ^237^ ^]^ While these measures can alleviate pain, improve function, or delay disease progression, they have significant limitations: long‐term use of nonsteroidal anti‐inflammatory analgesics (NSAIDs) causes gastrointestinal and cardiovascular side effects;^[^ ^238^ ^]^ although corticosteroids can reduce inflammation in the short term, they may accelerate cartilage degeneration and cannot be used repeatedly long‐term;^[^ ^239^ ^]^ injection therapies such as hyaluronic acid have limited efficacy and short duration; surgery is traumatic with limited prosthesis lifespan and cannot reverse pathological changes caused by local low‐grade chronic inflammation. Importantly, most existing treatments focus on symptom control or mechanical repair, struggling to precisely regulate local immune imbalance in joints (such as M1/M2 macrophage shift, Th17‐mediated inflammatory pathways), thus leaving significant gaps in suppressing chronic low‐grade inflammation and promoting tissue regeneration. In recent years, an increasing number of biomaterials targeting the specific immune microenvironment of OA have emerged. These materials not only possess excellent immunomodulatory functions but also demonstrate properties that promote cartilage regeneration, effectively advancing OA treatment progress.

**Figure 7:**
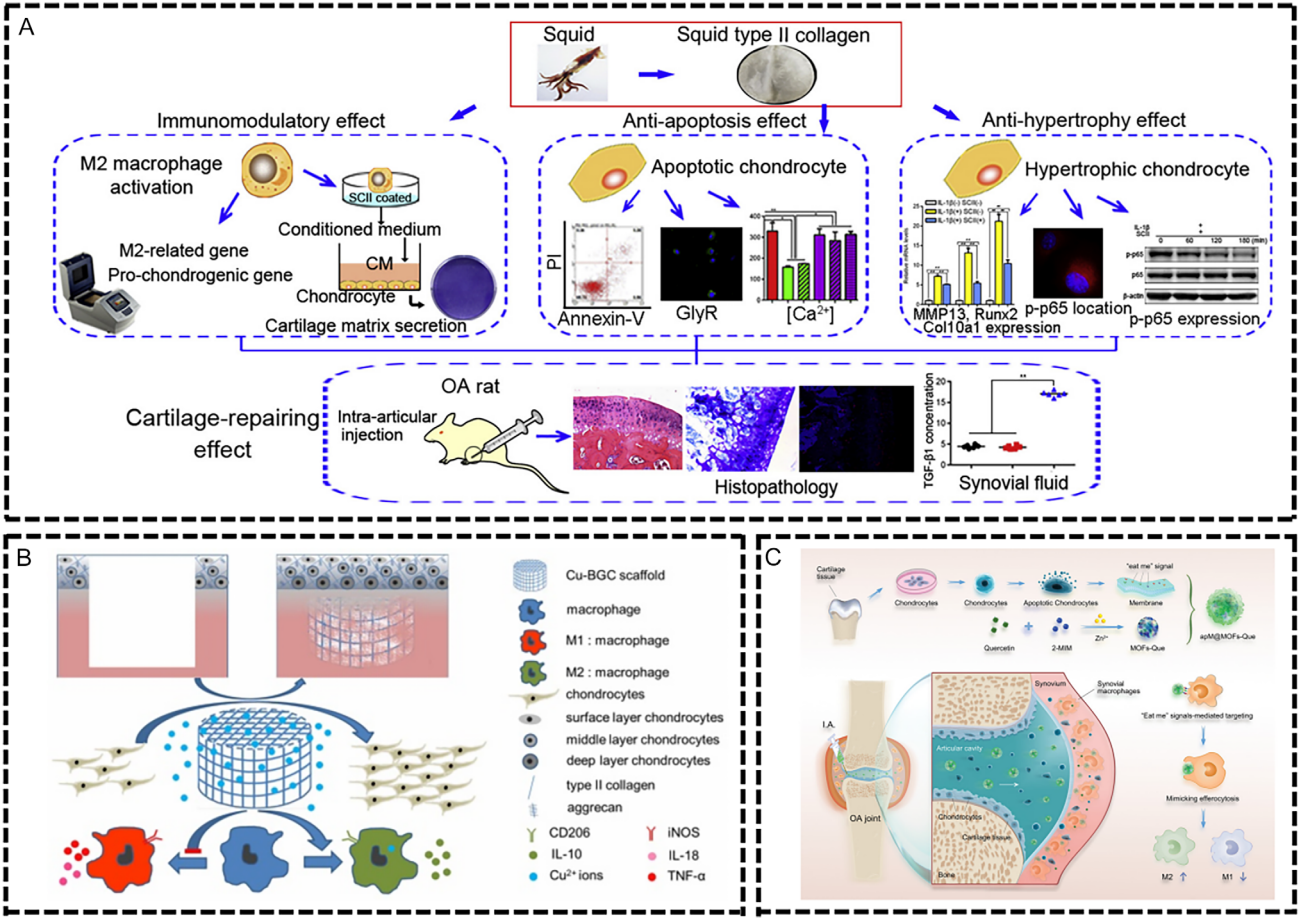
Biomaterials for the treatment of osteoarthritis (OA). A) Biomimetic squid cartilage Col II (SCII) promotes OA cartilage regeneration. SCII induced macrophage M2 polarization and activated macrophages to express chondrogenic genes (TGF‐β and IGF). Glycine in SCII activates glycine receptors on inflammatory chondrocytes and reduces intracellular calcium concentration, thereby effectively inhibiting chondrocyte apoptosis and MMP13 production, all of which have been verified in OA rat models. Reproduced with permission.^[^ ^241^ ^]^ Copyright 2018, Elsevier. B) Cu‐BGC scaffold for treating cartilage injury and reducing inflammatory response caused by osteoarthritis. Cu‐BGC can promote the proliferation and maturation of chondrocytes and the transformation of M0 macrophages into M2 macrophages, inducing anti‐inflammatory phenotype and regeneration of cartilage/bone interface. Reproduced with permission.^[^ ^244^ ^]^ Copyright 2019, Ivyspring International Publisher. C) A quercetin loaded metal‐organic framework (MOF) NPs platform coated with apoptotic chondrocyte membranes to treat OA by modulating synovial macrophage polarization. The platform can effectively induce macrophages to phagocytose nanoparticles and promote the polarization of synovial macrophages to anti‐inflammatory M2 macrophages. Reproduced with permission.^[^ ^248^ ^]^ Copyright 2022, Wiley‐VCH GmbH.

Immune cell activation, cytokine release, degradation, and remodeling of the extracellular matrix have an essential role in the progression of osteoarthritis. Creating a microenvironment that promotes cartilage repair is the key to curing OA. Decellularized cartilage ECM scaffolds have been reported to promote the invasion, migration, proliferation, and chondrogenic differentiation of BMSCs by inducing the polarization of M2 phenotype macrophages.^[^ ^240^ ^]^ In vitro experimental data showed that biomimetic squid cartilage Col II (SCII) induced M2 polarization of macrophages and activated macrophages to express pro‐chondrogenic genes (TGF‐β and IGF), improving the immune microenvironment around chondrocytes and promoting cartilage regeneration (**Figure** **7**A).^[^ ^241^ ^]^ Further studies showed that a bionic SGII hydrogel exhibited dual immunomodulatory effects on the pro/anti‐inflammatory phenotype of neutrophils and M1/M2 macrophage polarization. Subsequently, SGII promoted cartilage stem/progenitor cell chondrogenesis via the M2 macrophage‐mediated TGF‐β/Smad pathway.^[^ ^242^ ^]^

Jia et al. produced a temperature‐sensitive hydrogel (FPSOH) synthesized via a crosslinking between poly (salicylic acid)‐F127‐poly (salicylic acid) (FPS) and hyaluronic acid‐3‐hydroxyanthranilic acid (OH). 3‐Hydroxyanthranilic acid modulates the immune microenvironment of osteoarthritis through two mechanisms: One is to regulate the conversion of M1 macrophages to M2 macrophages, and the other is to inhibit the expression and release of inflammatory factors via the classical inflammatory signaling pathway such as nuclear factor kappa B (NF‐κB). In vitro studies have shown that FPSOH hydrogel can reprogram macrophage M1 phenotype to M2 phenotype and inhibit the expression of genes related to the NF‐κB signaling pathway.^[^ ^243^ ^]^

In addition, bioinorganic materials can also be used to regulate macrophage polarization to promote cartilage regeneration. Lin et al. produced a copper‐incorporated bioactive glass ceramics (Cu‐BGC) scaffold. Cu‐BGC has the capability to shift M0 macrophages to M2 macrophages. The expression of TNF‐α genes, IL‐18 genes in macrophages cultured with Cu‐BGC ionic products were downregulated, while IL‐10 genes were upregulated. It is noteworthy that Cu^2+^ combined with other materials may induce macrophages to form an M1 phenotype. In addition, Cu^2+^ can promote inflammation through its cytotoxic effect at higher concentrations (Figure 7B).^[^ ^244^ ^]^ Further studies are needed to explore a more suitable carrier and concentration. In addition, copper, as one of the essential components of many enzymes such as Cu‐Zn superoxide dismutase, has a powerful ability to trap free radicals in the nanoscale.^[^ ^245^ ^]^

There is increasing evidence that the polarization state of macrophages is susceptible to the physicochemical properties of biomaterials. Xue et al. synthesized a polycaprolactone (PCL) and eucalyptus gum (EUG) composite scaffold. They controlled the scaffold stiffness by adjusting the ratio of PCL and EUG, which in turn regulated macrophage polarization. High scaffold stiffness facilitated the polarization of M2 phenotype macrophages, but the exact mechanism still needs to be elucidated.^[^ ^246^ ^]^

Exosomes have an essential role in intercellular communication. 3D‐printed cartilage ECM/GelMA/exosome scaffolds promote cartilage regeneration by enhancing chondrocyte migration and polarizing synovial macrophages to the M2 phenotype.^[^ ^247^ ^]^ Nanoparticles targeting synovial macrophages are a potential therapeutic strategy. However, the therapeutic efficacy of injectable nanoparticles in the joint cavity is a great challenge due to the inefficient endocytosis of nanoparticles by synovial macrophages and the high clearance of nanoparticles by the joint cavity. Yang et al. developed an apoptotic chondrocyte membrane‐encapsulated, quercetin‐loaded metal‐organic framework (MOF) NPs, which can achieve active targeting of synovial macrophages (Figure 7C).^[^ ^248^ ^]^ On the one hand, the nanoparticles disguised with apoptotic chondrocyte membranes containing “eat‐me” signals can effectively induce macrophages to phagocytose the nanoparticles. On the other hand, the loaded quercetin can promote the polarization of synovial macrophages into anti‐inflammatory M2 macrophages.

Further research is still needed for the treatment of osteoarthritis. Immunomodulatory bioactive materials need to be more diversified, 3D, and precise. The development of new immune engineering materials at the cell, protein, and matrix levels is an urgent problem we need to solve.

### 4.4 Diabetic Wound

#### 4.4.1 The Immune Microenvironment of Diabetic Wound

Diabetic wounds are one of the serious complications faced by diabetic patients, characterized by delayed or nonhealing wound closure, severely affecting patients’ quality of life. Compared with normal wound healing processes, diabetic wounds exhibit persistent inflammatory responses, immune cell dysfunction, and cytokine network disturbances. The formation of this pathological immune microenvironment involves abnormal activation and dysfunction of multiple immune cells, including neutrophil overactivation, macrophage polarization imbalance, dendritic cell dysfunction, and abnormal T cell subset distribution. A thorough understanding of the characteristics of the diabetic wound immune microenvironment is of great significance for developing new therapeutic strategies.

In the immune response to skin injury, neutrophils are the first immune cells recruited to the wound site. They exert antimicrobial effects by producing reactive oxygen species (ROS), secreting matrix metalloproteinases (MMPs), and forming neutrophil extracellular traps (NETs). However, in chronic diabetic wounds, these originally protective mechanisms may instead lead to excessive tissue damage and delayed wound healing.^[^ ^249^ ^]^

One of the characteristic pathological changes in diabetic wounds is macrophage dysfunction. In the metabolic environment of hyperglycemia and elevated plasma free fatty acids, pro‐inflammatory cytokine expression is upregulated, driving macrophage polarization toward the M1 phenotype. M1 macrophages further secrete pro‐inflammatory factors such as TNF‐α, forming a positive feedback loop that maintains wounds in a chronic inflammatory state.^[^ ^250^ ^]^ Notably, fragments produced by M1 macrophage‐derived MMPs hydrolyzing extracellular matrix (ECM) can act as inducing factors, further regulating immune cell activity. Additionally, epigenetic changes in the hyperglycemic environment, including histone methylation and acetylation modifications, can also lead to increased pro‐inflammatory factor secretion.^[^ ^251^ ^]^

Interestingly, although M2 macrophages are generally considered to have anti‐inflammatory and prohealing effects, their role in diabetic wounds is more complex. Studies have shown that treating diabetic mice with M2 macrophages actually leads to delayed wound healing.^[^ ^252^ ^]^ Recent single‐cell transcriptome analysis has further revealed this paradox: healed diabetic foot ulcer (DFU) individuals have a higher proportion of M1 macrophages, while non‐healed individuals have more M2 macrophages,^[^ ^253^ ^]^ suggesting that the dynamic balance of macrophage subtypes rather than a single phenotype may be more important.

Other immune cells also play important roles in diabetic wound healing disorders. Langerhans cells, as specialized dendritic cells of the skin and mucosa, are responsible for antigen presentation and promoting keratinocyte proliferation. In diabetic wounds, Langerhans cells not only have impaired migration ability but also significantly reduced numbers, leading to delayed wound healing. T cell subset imbalance similarly exacerbates inflammatory responses: systemic inflammation restricts Treg cell migration to wound sites while promoting the infiltration of pro‐inflammatory Th17 cells.

Mast cells are recruited to wound sites through complement fragments C3a and C5a, subsequently releasing cytokines such as TNF‐α, VEGF, IL‐6, and IL‐8, participating in regulating angiogenesis and matrix remodeling. Although changes in mast cell numbers in diabetic wounds remain controversial, their degranulation function is significantly enhanced, leading to reduced release of mast cell‐derived VEGF and affecting neovascularization.^[^ ^254^ ^]^

Chemokine network disruption is another important mechanism in the chronicity of diabetic wounds. Pro‐inflammatory chemokines such as CCL2 and CXCL2 are significantly upregulated in diabetic wounds, closely associated with excessive infiltration of neutrophils and macrophages and sustained high expression of inflammatory factors such as IL‐1β and TNF‐α in late healing stages. Conversely, CXCL12, which has pro‐healing effects, is downregulated in the diabetic wound microenvironment.^[^ ^255^ ^]^ This alteration in chemokine expression profile further exacerbates the imbalance of the immune microenvironment, creating a vicious cycle unfavorable for wound healing.

#### 4.4.2 Current Treatments and the Immunomodulatory Effects of Biomaterials

Standard management of diabetic chronic wounds relies on glycemic control, local wound care, and infection control: including strict blood glucose management, compression or offloading therapy, debridement, maintaining a moist wound environment, antibiotics for infections, vascular reconstruction when necessary to improve local perfusion, and application of biological dressings or skin substitutes. Despite comprehensive management promoting healing in some wounds, diabetic wound healing rates remain unsatisfactory, with limiting factors including hyperglycemia‐induced immune dysfunction (neutrophil dysfunction, macrophage polarization imbalance), microvascular disease and ischemia, persistent oxidative stress, and advanced glycation end products (AGEs) deposition, leading to chronic inflammation and impaired tissue regeneration.^[^ ^256^ ^]^ Furthermore, traditional local therapies struggle to simultaneously correct ischemia, infection, abnormal inflammation, and oxidative environments, resulting in slow healing, recurrent infections, and significant amputation risks. Current treatment of diabetic foot ulcers still faces enormous challenges, and the emergence of immunomodulatory biomaterials brings new hope for accelerating wound healing. Among various biomaterials, hydrogels stand out due to their unique advantages and have become one of the most promising biomaterial systems in diabetic wound treatment.

**Figure 8:**
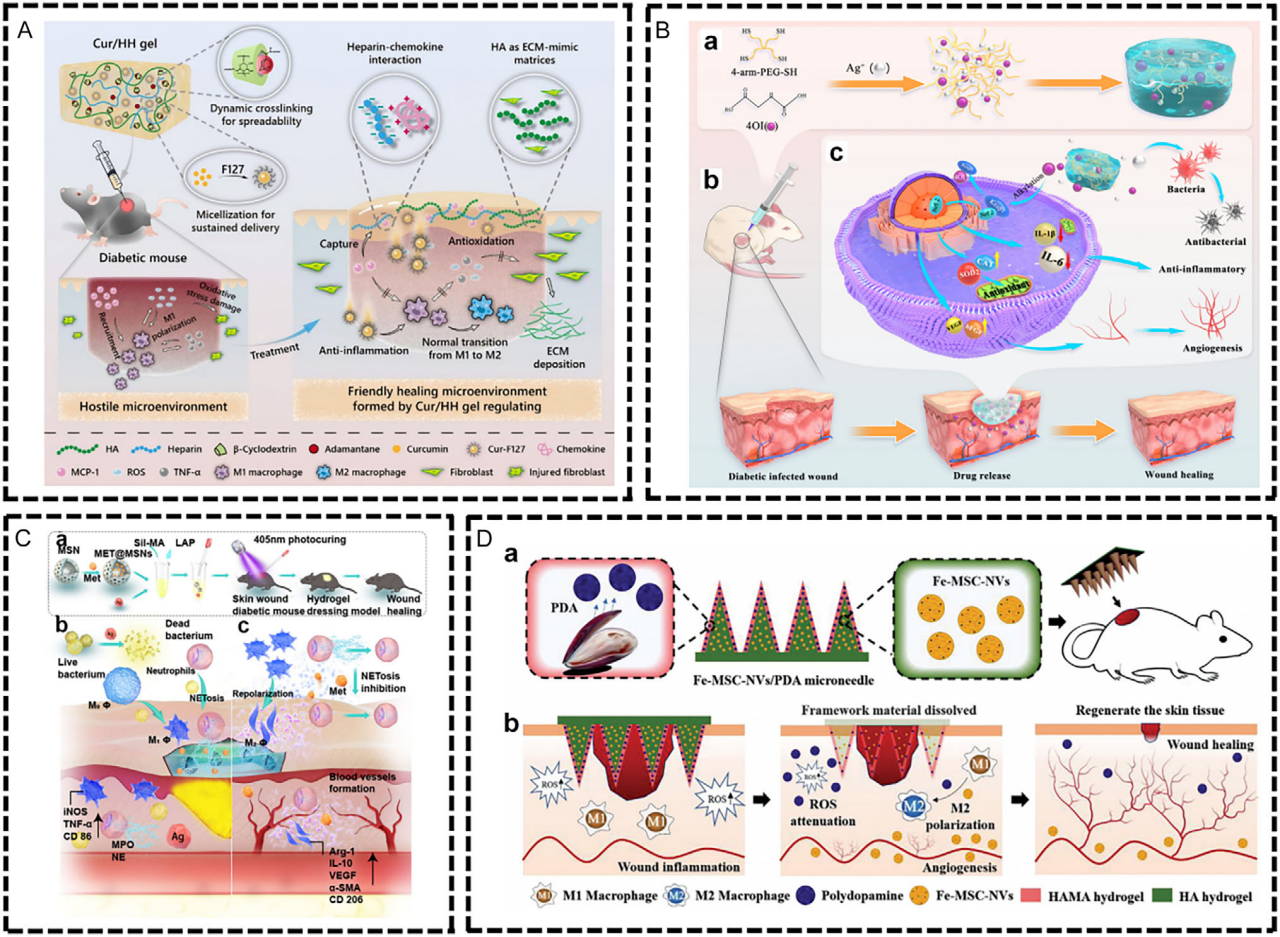
Biomaterials for diabetic wound treatment. A) A glycosaminoglycan‐based hydrogel delivery system modulates the wound microenvironment to promote chronic wound healing. Heparin captures inflammatory chemokines at the wound site, while hyaluronic acid mimics the function of ECM, and the hydrophobic drug curcumin is encapsulated in the hydrogel through micellar action, thus playing a good ROS scavenging ability and anti‐inflammatory activity. Reproduced with permission.^[^ ^257^ ^]^ Copyright 2022, American Chemical Society. B) The synthesis of 4OI@PEG hydrogels and their promotion of diabetic wound healing process. (a) Four‐arm‐PEG‐SH, 4OI and Ag constructed the dynamic coordinative hydrogels. (b) Intravenously injected hydrogel into diabetic wounds and sustained release of 4OI and Ag during wound repair. (c) 4OI@PEG hydrogel protects endothelial cells from ROS damage by activating Keap1‐Nrf2 antioxidant defense system and regulating mitochondrial polarization. Reproduced with permission.^[^ ^258^ ^]^ Copyright 2022, Elsevier. C) An injectable photocrosslinked wire hydrogel system promotes healing of diabetic wounds in orthopedic surgery through immune regulation. (a) Synthesis of Sil‐MA hydrogels (M@M‐Ag‐Sil‐MA) containing MET@MSNS and Ag NPs. (b) Initial controlled release of Ag NPs inhibits bacterial aggregation and creates a sterile microenvironment. (c) Dual‐controlled release of Met promotes wound healing through immune regulation of macrophages and NETs. Reproduced with permission.^[^ ^261^ ^]^ Copyright 2022, Springer Nature. D) Polydopamine‐modified microneedles were co‐encapsulated with Fe‐MSC‐derived nanovesicles to promote wound healing. (a) Schematic diagram of Fe‐MSC‐NVs/PDA MN patch for diabetic wound healing. (b) NPs is continuously released at the lesion to inhibit the ROS‐induced inflammatory response, and the combination of PDA NPs and Fe‐MSC‐NVs further promotes the polarization of M2 macrophages, thus inhibiting wound inflammation and promoting diabetic wound healing. Reproduced with permission.^[^ ^264^ ^]^ Copyright 2022, Wiley‐VCH GmbH.

Oxidative stress plays a crucial role in diabetic wound healing. The imbalance between oxidants and antioxidants leads to excessive production of reactive oxygen species (ROS), which in turn results in delayed wound healing. Zhang et al. developed a glycosaminoglycan‐based hydrogel drug delivery system to modulate the immune microenvironment of diabetic wounds (**Figure** **8**A).^[^ ^257^ ^]^ This study was validated in a streptozotocin (STZ)‐induced diabetic mouse model, where the hydrogel utilized heparin as a scaffold to capture inflammatory chemokines, while hyaluronic acid (HA) served as the extracellular matrix. Curcumin loaded in the hydrogel regulated oxidative stress by degrading ROS and exerted anti‐inflammatory effects. Itaconate is an endogenous metabolite with anti‐inflammatory and antioxidant activities. Polyethylene glycol (PEG) hydrogel loaded with 4‐octyl itaconate (4OI) (4OI@PEG hydrogel) protected endothelial cells from ROS damage by activating the Keap1‐Nrf2 antioxidant defense system and modulating mitochondrial polarization in both in vitro human umbilical vein endothelial cells (HUVECs) culture and diabetic rat wound models (Figure 8B).^[^ ^258^ ^]^

Another key characteristic of diabetic wounds is the imbalance in immune cell proportions, particularly the dysregulation of M1/M2 macrophage ratio. Rational regulation of macrophage polarization at different stages of wound healing is considered an important therapeutic approach for promoting diabetic wound healing. Studies have reported that multifunctional bone marrow mesenchymal stem cell‐derived exosomes (MSC‐Exo) loaded in carboxyethyl chitosan (CEC)‐dialdehyde carboxymethyl cellulose (DCMC) hydrogel (MSC‐Exos@CEC‐DCMC HG) can alleviate inflammation by promoting M1‐to‐M2 macrophage polarization in diabetic rat skin defect models, thereby synergistically regulating the immune microenvironment in chronic diabetic wounds.^[^ ^259^ ^]^

Natural extracts have tremendous potential in inducing macrophage polarization. Hyaluronic acid (HA) hydrogel loaded with paeoniflorin (PF) is evaluated in a full‐thickness dorsal skin wound model in STZ‐induced diabetic mice. In vitro experiments using bone marrow‐derived macrophages (BMDMs) confirm that PF can promote the transition from M1 to M2 macrophages. In vivo experiments demonstrate that this material accelerates wound healing, with histological analysis proving its superior supportive effects on angiogenesis and collagen deposition.^[^ ^260^ ^]^

The immune microenvironment of chronic diabetic wounds is extremely complex and exhibits spatiotemporal heterogeneity, thus necessitating the development of bioactive materials that can target different immune cells. In a recent study, a multifunctional methacryloyloxysilane hydrogel system, which synergistically encapsulates metformin‐loaded mesoporous silica microspheres and silver nanoparticles (AgNPs), promotes wound healing in diabetic mouse models (Figure 8C).^[^ ^261^ ^]^ This hydrogel system first releases AgNPs to exert antibacterial effects, and the resulting sterile microenvironment facilitates subsequent immunomodulation. This system not only regulates macrophage polarization from M1 to M2 phenotype but also inhibits the formation of neutrophil extracellular traps.

Additionally, Li et al. develop an injectable hydrogel based on hyaluronic acid‐dopamine (HA‐DA) and polydopamine (PDA)‐coated Ti_3_C_2_ MXene nanosheets. The oxyhemoglobin/hydrogen peroxide (HbO_2_/H_2_O_2_) system enables controlled oxygen release under near‐infrared (NIR) stimulation, improving hypoxic conditions. Meanwhile, MXene nanosheets and PDA coating synergistically scavenge excess reactive nitrogen species and ROS, maintaining redox homeostasis in wounds. Notably, HA‐DA molecules endow the hydrogel with the ability to regulate macrophages.^[^ ^262^ ^]^

Reports indicate that combined treatment of stem cell‐seeded cryogel/hydrogel biomaterials with acupuncture can produce synergistic immunomodulatory effects in diabetic rat models. Following acupuncture stimulation, infrared thermal imaging detects moderate increases in skin temperature, while ELISA analysis shows increased secretion of cytokines SDF‐1 and TGF‐β, with downregulation of pro‐inflammatory cytokines TNF‐α and IL‐1β.^[^ ^263^ ^]^ Ma et al. develop a core‐shell hyaluronic acid (HA) microneedle patch with needle tips encapsulating iron‐mesenchymal stem cell‐derived artificial nanovesicles (Fe‐MSC‐NVs) and polydopamine nanoparticles (PDA NPs) (Figure 8D).^[^ ^264^ ^]^ In STZ‐induced diabetic rat models, as the HA microneedle patch tips gradually degrade in the skin, PDA NPs and Fe‐MSC‐NVs are released into the wound. Immunohistochemical analysis reveals that this system can inhibit ROS‐induced inflammatory responses, reduce iNOS expression, and promote macrophage polarization toward the M2 phenotype.

Currently, the main strategies for immunomodulatory biomaterials used in diabetic wound treatment include maintaining redox homeostasis, regulating immune cell polarization, and releasing anti‐inflammatory mediators. These materials demonstrate promising therapeutic effects in various diabetic animal models. In the future, the functions of immunomodulatory materials need to be more diversified and precise. With the development of single‐cell sequencing technology, we have gained deeper insights into changes in immune cell subtypes in diabetic wounds. Recent studies indicate that epigenetic regulatory changes in immune and structural cells within wounds may affect cell phenotypes and healing processes, particularly pronounced in pathological conditions such as diabetes.^[^ ^265^ ^]^ Epigenetic regulation brings new hope for diabetic wound healing. Furthermore, as most immunomodulatory biomaterials currently remain in preclinical research stages, improving biosafety, optimizing the predictive value of in vitro and in vivo models, and reducing production costs are critical issues that require urgent attention for advancing clinical translation.

### 4.5 Spinal Cord Injury (SCI)

#### 4.5.1 The Immune Microenvironment of SCI

After spinal cord injury (SCI), the homeostasis of the spinal cord microenvironment is rapidly disrupted, triggering a cascade of pathophysiological changes. A systematic understanding of stage‐specific pathology and its constraints on functional reconstruction is fundamental to devising effective therapeutic strategies. By etiology, SCI is classified as traumatic or nontraumatic: Traumatic SCI is more common and typically induced by external forces, whereas nontraumatic SCI is often caused by tumor compression, vascular ischemia, or congenital malformations.^[^ ^266^, ^267^ ^]^

From a pathological progression perspective, SCI consists of two components: primary injury and secondary injury.^[^ ^268^ ^]^ Primary injury involves mechanical destruction, including contusion, compression, laceration, or transection, directly destroying neural and vascular structures, leading to acute cellular dysfunction and death.^[^ ^269^ ^]^ More critically, primary injury disrupts the blood‐spinal cord barrier (BSCB), thereby triggering a cascade of secondary injury reactions involving extensive biochemical, mechanical, and physiological changes.^[^ ^270^ ^]^ During the secondary process, peripheral blood cell infiltration and massive cytokines released by damaged or necrotic parenchymal cells collectively amplify the inflammatory response, causing further death of neurons and glial cells, and inducing significant imbalance in immune and neural network remodeling.

Different stages exhibit microenvironmental imbalance at tissue, cellular, and molecular levels. At the tissue level, manifestations include hemorrhage and ischemia, glial scar formation, demyelination and remyelination, with these changes inhibiting axonal regeneration and neural circuit reconstruction in the injury zone through multiple pathways.^[^ ^271^ ^]^ At the cellular level, changes include astrocyte activation, endogenous neural stem cell (NSCs) differentiation, and macrophage infiltration. The inflammatory response is intricately driven by multiple cell types and various mediators: Myelin debris can regulate macrophage activation, causing bone marrow‐derived macrophages (BMDMs) to shift from M2 to M1 phenotype accompanied by lipid accumulation, thereby shaping the pathological process.^[^ ^272^ ^]^ Interactions between neutrophils and extracellular matrix (ECM) components inhibit axonal regeneration, synaptic plasticity, and remyelination, while releasing various pro‐inflammatory mediators that exacerbate tissue destruction, with matrix metalloproteinases (MMPs) and chondroitin sulfate proteoglycans (CSPGs) being particularly crucial.^[^ ^273^ ^]^

In human SCI, lymphocyte infiltration is relatively low and predominantly consists of CD8+ T cells. CD8+ T cells damage the central nervous system (CNS) by releasing perforin and increase BSCB permeability, thereby promoting further entry of inflammatory cells and cytokines.^[^ ^274^ ^]^ Representative inflammatory factors include TNF‐α, IL‐1β, and IL‐6, which can mobilize and expand infiltration of microglia, peripherally derived macrophages (PDM), and neutrophils, while inducing secondary mediator release, forming a vicious cycle.^[^ ^275^ ^]^ Although inflammation intensity is influenced by initial stimulation and injury severity, primary injury is typically sufficient to trigger excessive inflammatory activation, leading to further cell death and tissue damage.^[^ ^276^ ^]^

Excitotoxicity is another core destructive process after SCI. Elevated glutamate in the lesion overactivates neighboring neurons, inducing intracellular Ca^2+^ overload and the excessive generation of reactive oxygen species. These highly reactive molecules attack cell membranes and intracellular structures, culminating in the death of neurons and oligodendrocytes and further impairing conduction and repair capacity.^[^ ^277^ ^]^

Overall, neuroinflammation after SCI is double‐edged, with outcomes hinging on the activation states of immune cells such as microglia and macrophages. M1 macrophages tend to amplify inflammation and inhibit regeneration, whereas M2 macrophages attenuate inflammatory infiltration, promote axonal growth, and support functional recovery. Thus, driving macrophage polarization toward the M2 phenotype is considered a promising therapeutic strategy warranting deeper mechanistic investigation and clinical translation.^[^ ^278^ ^]^

#### 4.5.2 Current Treatments and the Immunomodulatory Effects of Biomaterials

Clinical management of spinal cord injury includes acute‐phase neurosurgical stabilization, decompression surgery, spinal fixation, as well as rehabilitation support and complication management.^[^ ^279^ ^]^ Local or systemic neuroprotective drugs and hemodynamic management are also used for acute‐phase support. Long‐term rehabilitation includes physical therapy, functional training, and symptomatic treatment for complications such as pain and muscle spasms. Current treatment limitations primarily include: severe secondary damage following primary mechanical injury (inflammatory response, oxidative stress, blood‐brain barrier/blood‐spinal cord barrier disruption, apoptosis, etc.), with these pathological processes rapidly expanding the injury area, while existing clinical methods struggle to precisely inhibit harmful inflammation in time and space while maintaining or promoting immune support necessary for regeneration;^[^ ^269^ ^]^ limited neuronal and axonal regeneration, glial scar formation, and inhibitory microenvironment result in very limited functional recovery.^[^ ^280^ ^]^ Cell or stem cell transplantation is currently one of the most promising strategies for treating SCI.^[^ ^281^ ^]^ However, inflammatory responses induced by the immune microenvironment directly affect transplanted cell survival, and immune cells, as primary targets for inflammation regulation, can be modulated through biomaterials and loaded bioactive molecules. Biomaterials, with their excellent biocompatibility, tunable degradability, and low immunogenicity, are becoming important platforms for immunomodulation and inflammation suppression.^[^ ^282^ ^]^

**Figure 9:**
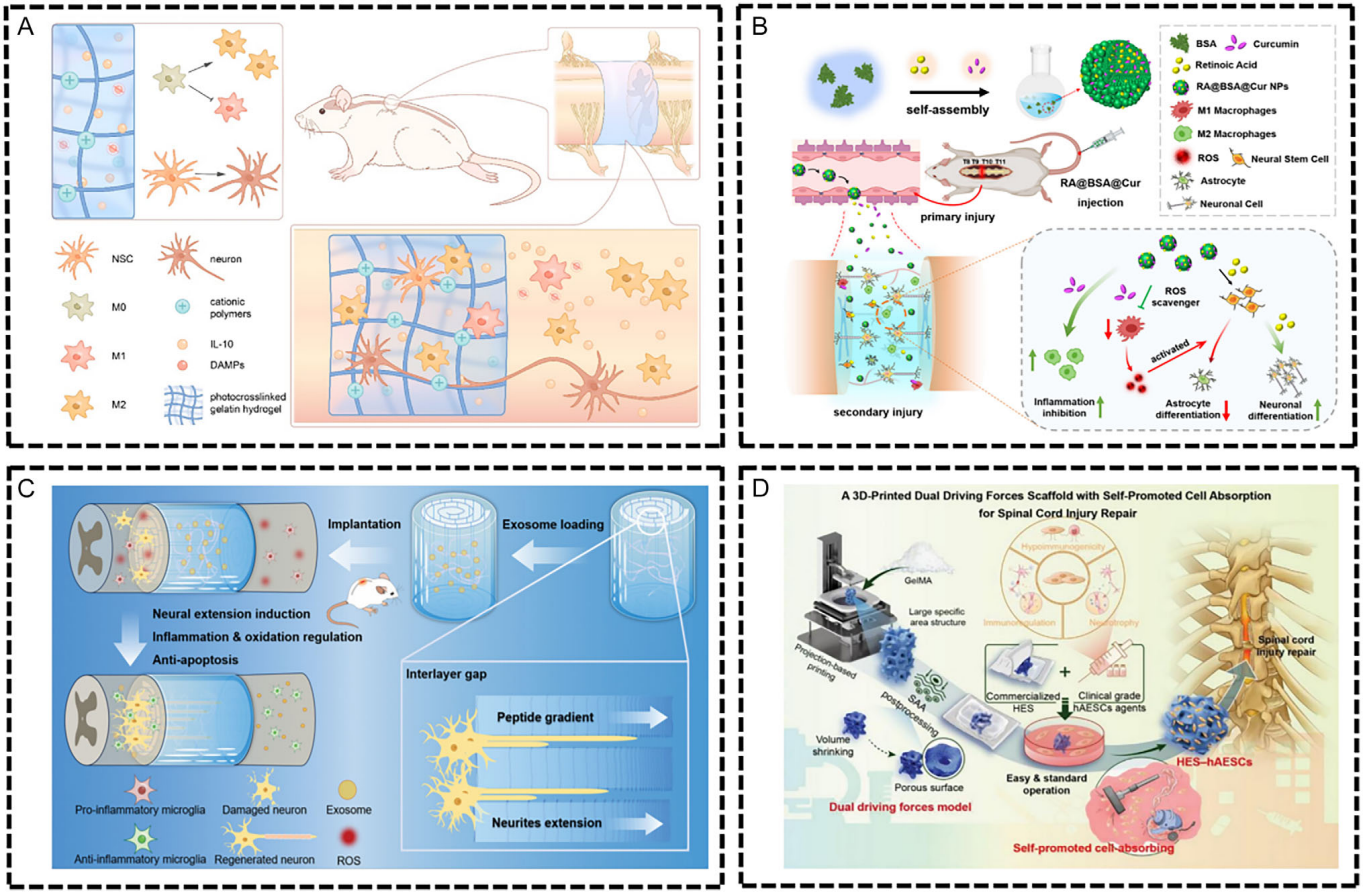
Biomaterials for spinal cord injury (SCI) treatment. A) A hydrogel capable of scavenging DAMP and releasing IL‐10 can promote nerve regeneration and motor function recovery after spinal cord injury. Photocrosslinked gelatin hydrogels are modified with cationic polymer poly (amidoamine) and anti‐inflammatory cytokine IL‐10. The scaffolds promote M2 macrophage polarization and neuronal differentiation in vitro. Stents injected into spinal cord injury sites in mice scavenged DAMP, reduced inflammation, and enhanced tissue remodeling and nerve regeneration. Reproduced with permission.^[^ ^285^ ^]^ Copyright 2022, Elsevier. B) A RA@BSA@Cur NPs platform for repair of transsectional spinal cord injury, anti‐inflammatory and neuroprotective. Retinal acid (RA) and curcumin (Cur) were co‐loaded into bovine serum albumin (BSA) by self‐assembly method to form RA@BSA@Cur NPs, which corrected the imbalance of macrophages, inhibited the release of inflammatory factors, promoted the production of neurons and axons, and prevented the proliferation of scar tissue. Reproducedwith permission.^[^ ^287^ ^]^ Copyright 2022, Elsevier. C) Functionalized scaffolds with helical shapes and peptide gradients can significantly support and promote the extension of neurites along the predetermined direction within the interlaminar spaces. Msc‐derived exosomes can be loaded into scaffolds to achieve multifactorial treatment for SCI rehabilitation by exerting inflammatory and oxidative regulation as well as anti‐apoptotic effects. Reproduced with permission.^[^ ^290^ ^]^ Copyright 2024, Wiley‐VCH GmbH. D) The dual‐driving force model HES‐hAESCs for SCI repair. The Gelatin‐methacryloyl (GelMA) scaffold with a high specific surface area was dehydrated through super‐absorption and adhesion (SAA) post‐treatment to form a dual‐driving force model, making HES a SCI repair delivery tool with a high hAESCs load. Reproduced with permission.^[^ ^292^ ^]^ Copyright 2023, Wiley‐VCH GmbH.

Hydrogel provides a microenvironment for resident cells after SCI to physically reconstitute the ECM and connect SCI‐associated damage gaps to guide neural axon growth. Liu et al. performed CS‐CA‐DA (chitosan‐citric acid‐dopamine) hydrogel scaffold implantation therapy for SCI repair, promoting cell survival, immunomodulation, macrophage polarization to M2 phenotype, and axonal regeneration.^[^ ^283^ ^]^ Han et al. used TUDCA‐hydrogel (TC gel) obtained from crosslinking HA with taurine deoxycholic acid (TUDCA) in SCI and found that the expression levels of pro‐inflammatory cytokines IL‐1 β, IL‐6, IFN‐γ, and TNF‐α were reduced.^[^ ^284^ ^]^ The Shen's team developed a DAMP clearing hydrogel scaffold with continuous anti‐inflammatory IL‐10 release, which inhibited the M1 polarization of macrophages and microglia after SCI, and improved the neural differentiation of neural stem cells (NSCs) (**Figure** **9**A).^[^ ^285^ ^]^

Nanoparticles with excellent biocompatibility and high drug loading efficiency constitute a modular and multimodal platform that can be specifically designed to overcome physiological barriers to the delivery of drugs to the central nervous system (CNS).^[^ ^286^ ^]^ In particular, the physicochemical properties of nanoparticles, including their size, surface chemistry, and composition, can be fine‐adjusted to achieve effective aggregation in damaged regions. Gao et al. constructed retinoic acid (RA) and curcumin (Cur) with bovine serum albumin (BSA), which effectively promoted the outgrowth of PC12 cells and the neural differentiation of bone marrow mesenchymal stem cells (Figure 9B).^[^ ^287^ ^]^ Jeong et al. found that after intravenous injection of NPs after spinal cord injury, blood monocytes were not significantly transported to the inflammatory area, which reduced M1 macrophage polarization and microglial cell activation.^[^ ^288^ ^]^ Jaffer The team designed a nanoparticle that encapsulates antioxidant enzymes to reduce the ROS produced by dysfunctional cell mitochondria at the damage site and combat the destruction of redox balance after SCI.^[^ ^289^ ^]^

In order to promote neural repair after SCI, functional scaffolds are attracting widespread attention. Huang et al. designed a scaffold with a helical structure and gradient peptide modification, which could significantly induce nerve extension in vitro and significantly promote functional recovery and nerve repair in vivo, and had excellent drug loading capacity (Figure 9C).^[^ ^290^ ^]^ Studies have shown that the combination of decellularized extracellular matrix (dECM) scaffolds with adipose‐derived stem cells (ADSCs) can promote neural regeneration and functional recovery in mice with spinal cord injury by regulating the Wnt/β‐catenin signaling pathway.^[^ ^291^ ^]^ Meanwhile, Qiu et al. developed a novel cell delivery platform of hyper expansion scaffolds (HES) loaded with high human amniotic epithelial stem cells (hAESC). This platform promoted the efficient absorption of loaded cells through a dual‐driving force model and facilitated the recovery of spinal cord function by reducing neuroinflammation and improving the microenvironment (Figure 9D).^[^ ^292^ ^]^

Due to the importance and vulnerability of CNS, the regulation of the immune microenvironment of SCI should be very careful, and the delivery process using bioactive materials is an important challenge for the treatment of SCI.

### 4.6 IVDD

#### 4.6.1 The Immune Microenvironment of IVDD

The intervertebral disc (IVD) is a crucial spinal component with a unique structure comprising the central nucleus pulposus (NP), peripheral annulus fibrosus (AF), and cartilage endplates (CEP). As one of the body's largest avascular tissues, the IVD presents a harsh microenvironment characterized by hypoxia, nutrient deficiency, acidity, high osmotic pressure, and mechanical loading.^[^ ^293^, ^294^, ^295^, ^296^, ^297^ ^]^ These conditions cause chronic cellular damage and metabolic imbalance, ultimately leading to progressive cellular dysfunction.

Intervertebral disc degeneration (IVDD) represents pathological aging influenced by mechanical stress, oxidative stress, and inflammatory cytokines, which induce disc cell apoptosis and necrosis.^[^ ^298^ ^]^ The IVDD immune microenvironment changes involve both endogenous and exogenous factors.^[^ ^299^ ^]^ Endogenous factors include microcrystal formation and ECM degradation products that act as DAMPs, activating inflammatory pathways.^[^ ^300^ ^]^ Exogenous factors involve mechanical loading‐induced microfractures or herniation, exposing nucleus pulposus to the immune system and triggering inflammatory responses.^[^ ^301^ ^]^

Autoimmune activation is a core pathological mechanism in IVDD, with inflammatory mediators playing key roles.^[^ ^302^ ^]^ Progressive nucleus pulposus dysfunction constitutes the main pathological basis.^[^ ^303^ ^]^ Persistent inflammation creates a harmful microenvironment that accelerates ECM degradation and recruits inflammatory cells through positive feedback mechanisms, exacerbating disease progression.

Oxidative stress drives IVDD through ROS production from mitochondrial dysfunction, triggering cellular senescence and inflammation.^[^ ^304^ ^]^ Macrophages and neutrophils are early responders in acute IVDD,^[^ ^305^ ^]^ with macrophage infiltration rapidly amplifying inflammation. Immune cells regulate the inflammatory environment through cytokine secretion, influencing tissue repair.^[^ ^306^ ^]^ Meanwhile, other innate immune cells, such as dendritic cells and mast cells, respond rapidly and secrete pro‐inflammatory cytokines after macrophage infiltration. However, they also assist in the recovery of IVDD by stimulating angiogenesis, recruiting adaptive immune cells and becoming resident immune cells.^[^ ^307^ ^]^

Key inflammatory cytokines including TNF‐α, IL‐1α/β, IL‐6, and IL‐17 are elevated in IVDD, promoting matrix degradation and chemokine production.^[^ ^308^, ^309^ ^]^ This creates an inflammatory cascade that attracts more immune cells.^[^ ^310^ ^]^ Conversely, anti‐inflammatory factors like IL‐4 and IL‐10, produced by activated monocytes and M2 macrophages,^[^ ^311^ ^]^ help maintain immune balance by limiting excessive inflammation and creating conditions for tissue repair.

#### 4.6.2 Current Treatments and the Immunomodulatory Effects of Biomaterials

Conventional management of intervertebral disc degeneration primarily consists of conservative treatment, including nonsteroidal anti‐inflammatory drugs, physical therapy, mechanical disc decompression, and surgical intervention when conservative therapy fails (discectomy, fusion, or artificial disc replacement).^[^ ^312^ ^]^ While conservative treatment can alleviate symptoms, it cannot reverse the degenerative process; although surgery can relieve nerve compression symptoms, it carries risks of complications, compensatory degeneration of adjacent segments, and postoperative pain. The intervertebral disc is an immune‐privileged zone with avascular characteristics, where immune exposure during degeneration, persistent inflammatory cell infiltration, oxidative stress, and cellular senescence collectively create an irreversible and harmful microenvironment.^[^ ^304^ ^]^ Existing treatment strategies typically focus on mechanical decompression or pain control, lacking immunological interventions targeting chronic inflammation within the disc, thus having limited capacity for fundamental pathological repair, with recurrence and long‐term functional decline remaining prevalent. Utilizing biomaterials to construct local drug delivery systems for IVDD treatment has gained increasing attention, as this helps achieve sustained drug release and reduces the need for repeated injections.^[^ ^313^ ^]^ Current approaches mainly employ delivery methods such as nanoparticles and hydrogels, establishing drug delivery platforms with good biocompatibility and high safety to improve therapeutic outcomes.^[^ ^314^ ^]^

**Figure 10:**
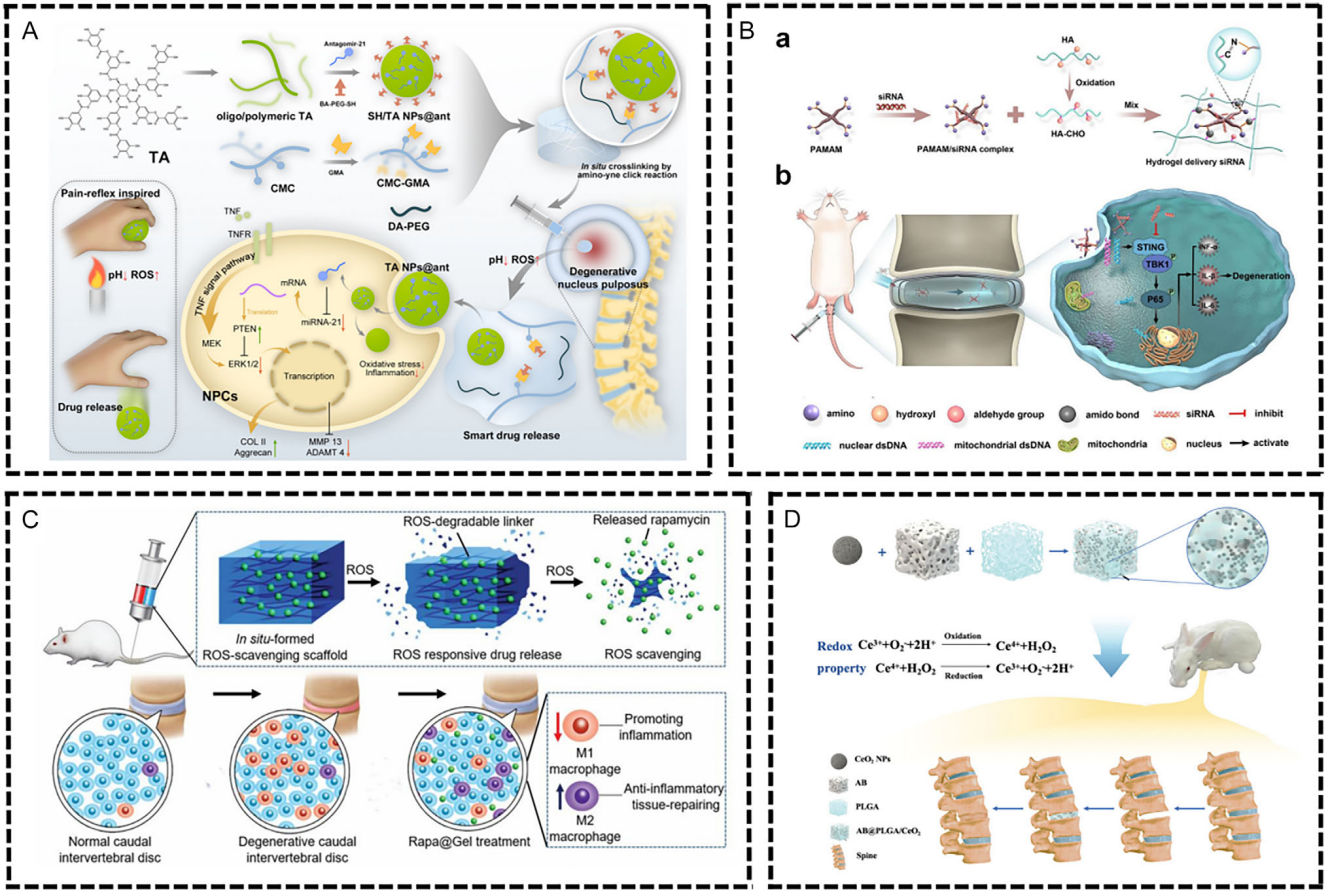
Biomaterials for the treatment of intervertebral disc degeneration (IVDD). A) A polyphenol nanosphere encapsulated hydrogel gene delivery system for repairing degenerative nucleus pulposus. By integrating antagomir‐21 and functional tannic acid (TA) NPs into TA NPs@ant, the hydrogel was mainly composed of glycidyl methacrylate (GMA) modified carboxymethyl chitosan (CMC‐GMA). The inflammatory TA NPs@ant is delivered first from the hydrogel and then intracellular antagomir‐21 from TA NPs@ant to achieve ROS clearance, anti‐inflammatory effects and regulation of ECM metabolic homeostasis in nucleus pulposus. Reproduced with permission.^[^ ^317^ ^]^ Copyright 2023, Elsevier. B) An injectable self‐healing hydrogel with siRNA delivery properties for sustained STING silencing and enhanced disc degeneration therapy. (a) The hydrogel consists of hyaluronic acid (HA) and polyaminamine (PAMAM) dendrimers. Aldehyde functionalized HA (HA‐CHO) was synthesized by oxidation, and PAMAM/siRNA was prepared to obtain siRNA@HPgel. (b) The use of manufactured hydrogels to silence the IVD denaturation therapy process through the sustained STING pathway. STING recruits tank‐bound kinase 1 (TBK1), and TBK1 phosphorylates interferon regulatory factor 3 (IRF3) and NF‐κB to induce the production of type I interferon (IFN) and pro‐inflammatory cytokines. These include TNF‐α, IL‐1β, and IL‐6. Reproduced with permission.^[^ ^321^ ^]^ Copyright 2023, Elsevier. C) Ros‐reactive hydrogels loaded with Rapa regulate the IVDD immune microenvironment by promoting the phenotypic polarization of anti‐inflammatory M2‐like macrophages. Reproduced with permission.^[^ ^323^ ^]^ Copyright 2019, Wiley‐VCH GmbH. D) Preparation of AB@PLGA/CeO_2_ and its antioxidant mechanism and intervertebral fusion effect in New Zealand white rabbits. Reproduced with permission.^[^ ^325^ ^]^ Copyright 2024, Wiley‐VCH GmbH.

Nanotechnology is at the forefront of medical diagnostics, imaging, and therapeutic drug delivery. It has the potential to enhance the control of drugs and materials by changing the size of them, and expand in the field of IVDD treatment.^[^ ^315^ ^]^ Gorth et al. showed that IL‐1ra‐PLGA microspheres can be released cumulative over 35 days and could comprehensively attenuate the effects of IL‐1β‐induced inflammation, and potentially treat inflammation‐mediated IVDD.^[^ ^316^ ^]^ Wang et al. developed a polyphenol nanosphere‐coated delivery system to achieve local sustained release of anti‐miRNA 21 inhibitors in IDD, inhibit mitogen‐activated protein kinase/extracellular signal‐regulated kinase (MAPK/ERK) signaling pathway, clear endogenous ROS and reduce TNF‐α expression, showing significant anti‐inflammatory and regenerative effects (**Figure** **10**A).^[^ ^317^ ^]^ Yang et al. constructed a polydopamine nanoparticle (PDA NPs) to reduce the inflammatory response caused by IVDD by scavenging reactive oxygen species, chelating Fe^2+^, and inhibiting glutathione peroxidase 4 (GPX 4) ubiquitination.^[^ ^318^ ^]^

Hydrogels are considered as important materials for the treatment of IVDD for their high water content, good biocompatibility, 3D network structure, and suitable biomechanical properties.^[^ ^319^ ^]^ An anti‐quercetin containing quercetin and quercetin effectively weakened the expression of NP cell senescence markers and restored metabolic homeostasis.^[^ ^320^ ^]^ Chen et al. used the cytosolmic dsDNA accumulation and stimulator of interferon genes (STING)‐NF‐κB pathway activation as the siSTING delivery hydrogel of aldehyde hyaluronic acid (HA‐CHO) and poly(amidoamine) PAMAM/siRNA complex to intervene with the abnormal STING signaling of IVD degeneration treatment, which significantly alleviated IVD inflammation and slowed IVD degeneration (Figure 10B).^[^ ^321^ ^]^ Liu et al. designed an injectable self‐repairing multifunctional hydrogel made with dopamine functionalized gelatin (GelDA) and borax‐coupled aldehyde‐modified chondroitin sulfate (Borax‐ACS), loaded with extracellular vesicle‐glutaredoxin 3 (EVs‐GLRX3), proving to alleviate mitochondrial damage, restore extracellular matrix deposition and reduce the senescence of NP cells.^[^ ^322^ ^]^

The development of tissue engineering is increasingly being highly anticipated for the treatment of IVDD. Bai et al. developed a rapamycin‐loaded ROS‐clearing scaffold (Rapa@Gel) that promotes anti‐inflammatory M2 macrophage phenotypic polarization, reduces intervertebral disc inflammatory response and promotes regeneration (Figure 10C).^[^ ^323^ ^]^ Choy et al. constructed a biomimetic mechanically functional biphasic scaffold composed of collagen and glycosaminoglycans (GAG) that mimics the structure and function of intervertebral discs, showing promise as a replacement for the nucleus pulposus.^[^ ^324^ ^]^ Li et al. developed a composite scaffold material AB@PLGA/CeO_2_, in which cerium dioxide nanoparticles (CeO_2_ NPs) were loaded onto the surface of allograft bone (AB). This material exhibits excellent antioxidant and radical scavenging effects, effectively enhancing bone regeneration and promoting collagen fiber formation and mineralization. Moreover, transcriptome sequencing revealed that it promotes osteogenic differentiation by regulating the extracellular matrix and the PI3K‐Akt signaling pathway (Figure 10D).^[^ ^325^ ^]^

At present, the treatment of bioactive materials for intervertebral disc degeneration is still under extensive research and testing. This limits the selection of active materials and delivery molecules due to the harsh and vulnerable nature of the disc immune microenvironment. Adverse reactions and efficacy limitations seen in clinical trials have led to the delayed approval of the clinical application of treatment options. Therefore, combining effective disease‐relieving drugs with safe administration strategies is essential for achieving disc regeneration.

## 5 Current Challenges and Future Perspectives

At present, the clinical translation of biomaterials for chronic inflammatory diseases of bone and soft tissues faces multiple challenges, calling for systematic progress from materials design and delivery strategies to long‐term safety assessment and industrial feasibility.

First, lesion sites and their inflammatory microenvironments exhibit marked heterogeneity, imposing higher demands on the stability and biocompatibility of biomaterials.^[^ ^326^ ^]^ Materials must maintain mechanical and chemical stability and controlled release under pathological conditions such as acidity, oxidative stress, or high enzymatic activity, while avoiding further immune activation or cytotoxicity.

Second, precise, sustained, and site‐specific delivery systems are key to therapeutic efficacy. Given the dense structure and limited blood supply of bone tissue, and the vascular abnormalities often accompanying inflamed soft tissues, conventional systems struggle to balance penetration with local retention.^[^ ^327^ ^]^ Future strategies should adopt multiscale delivery, such as integrated platforms that combine nanocarriers with local scaffolds/hydrogels, as well as targeted ligands or immune cell‐guided delivery, to increase drug/biologic accumulation at lesions, extend release profiles, and reduce systemic exposure and adverse effects.^[^ ^328^ ^]^

Third, evidence on long‐term efficacy and safety remains insufficient. Current work is mostly short‐term animal or in vitro studies, with limited data on immune tolerance under chronic use, toxicology of long‐term degradation products, and interactions with comorbidities. In recent years, the convergence of immunology and materials science has led a number of biomaterial platforms designed for immune modulation to enter clinical trials or obtain regulatory approval for clinical use. Clinical efforts are primarily focused on: surface modification of orthopedic implants for local anti‐infection or immune modulation;^[^ ^329^ ^]^ immunemodulatory dressings and hydrogels for acute and chronic wounds;^[^ ^330^ ^]^ material‐based delivery systems for immune cells or therapeutics administered locally or systemically;^[^ ^331^, ^332^ ^]^ and multimodal therapy coupled with physical stimulation.^[^ ^333^, ^334^ ^]^ To promote greater clinical translation, there is an urgent need to establish comprehensive long‐term animal models and standardized immunotoxicological evaluation systems, as well as to conduct early human cohort studies.

In addition, advanced biotechnologies and engineering strategies can markedly broaden therapeutic boundaries. Engineered cell therapies and gene therapies can directly modulate the lesion's immune microenvironment or promote tissue regeneration.^[^ ^335^, ^336^ ^]^ Combining these cell/gene therapies with controllably degradable biomaterial carriers can enable controlled release, cell localization, and microenvironment‐friendly modulation, thereby enhancing efficacy and reducing systemic side effects. At the same time, these approaches raise higher requirements regarding immunogenicity, genetic safety, control of long‐term expression, and regulatory compliance.

The clinical translation of immunomodulatory biomaterials requires deep integration of multidisciplinary collaboration and sustainable development strategies. Fields including materials science, immunology, and pharmacokinetics should form a tight collaborative chain with regulatory agencies and industry partners, establishing unified quality control standards and safety assessment systems from the early stages of research and development. During industrialization, priority should be given to selecting scalable, cost‐controllable raw materials, and processing routes, while rigorously evaluating organic solvent usage, waste management, and the ecotoxicology of degradation products to achieve both clinical and environmental sustainability. By strengthening clinical pathway design and cost‐effectiveness analysis, the translation cycle from laboratory to clinic can be shortened, ultimately ensuring that patients with chronic inflammatory diseases have timely access to safe, effective, and economically accessible immunomodulatory therapies.

## 6 Conclusion

The occurrence and development of chronic inflammatory diseases are closely related to immune microenvironment imbalance, involving abnormal polarization of various immune cells, imbalance of pro‐inflammatory/anti‐inflammatory factors, and pathological remodeling of extracellular matrix. In recent years, immunomodulatory biomaterials have provided innovative strategies for treating chronic inflammatory diseases of bone and soft tissue by targeting immune cell phenotype regulation, reshaping cytokine networks, and maintaining microenvironmental homeostasis. We systematically explore the mechanisms of biomaterials such as nanoparticles, hydrogels, and scaffolds in regulating immune responses and demonstrate their remarkable therapeutic efficacy in chronic inflammatory diseases through multimodal synergy. Although significant progress has been made in related research, clinical translation still faces numerous bottlenecks. Looking forward, coordinated advancement is needed in intelligent material design, precise delivery, long‐term safety assessment, and industrialization pathways. The sustainable development of immunomodulatory biomaterials relies on interdisciplinary in‐depth collaboration and standardization system construction, including the establishment of unified evaluation criteria, stratified clinical pathway planning, and systematic accumulation of evidence‐based medical data.

## Conflict of Interest

The authors declare no conflict of interest.
